# Early Pleistocene faunivorous hominins were not kleptoparasitic, and this impacted the evolution of human anatomy and socio-ecology

**DOI:** 10.1038/s41598-021-94783-4

**Published:** 2021-08-09

**Authors:** Manuel Domínguez-Rodrigo, Enrique Baquedano, Elia Organista, Lucía Cobo-Sánchez, Audax Mabulla, Vivek Maskara, Agness Gidna, Marcos Pizarro-Monzo, Julia Aramendi, Ana Belén Galán, Gabriel Cifuentes-Alcobendas, Marina Vegara-Riquelme, Blanca Jiménez-García, Natalia Abellán, Rebeca Barba, David Uribelarrea, David Martín-Perea, Fernando Diez-Martin, José Manuel Maíllo-Fernández, Antonio Rodríguez-Hidalgo, Lloyd Courtenay, Rocío Mora, Miguel Angel Maté-González, Diego González-Aguilera

**Affiliations:** 1 Institute of Evolution in Africa (IDEA), Alcalá University, Covarrubias 36, 28010 Madrid, Spain; 2 Area of Prehistory (Department History and Philosophy), University of Alcalá, 28801 Alcalá de Henares, Spain; 3 Regional Archaeological Museum of Madrid, Plaza de las Bernardas s/n, Alcalá de Henares, Spain; 4 Osteoarchaeological Research Laboratory, Department of Archaeology and Classical Studies, Stockholm University, 106 91 WallenberglaboratorietStockholm, Sweden; 5 Department of Archaeology and Heritage Studies, University of Dar Es Salaam, P.O. Box 35050, Dar es Salaam, Tanzania; 6 The Luminosity Lab, Arizona State University, Tempe, AZ USA; 7 Paleontology Unit, National Museum of Tanzania in Dar Es Salaam, Robert Shaban St, P.O. Box 511, Dar es Salaam, Tanzania; 8 UMR5608, CNRS TRACES, Université Toulouse Jean-Jaurès, Maison de La Recherche, 5 allées Antonio Machado, 31058 Toulouse Cedex 9, France; 9 Artificial Intelligence Department, Universidad Nacional de Educación a Distancia, UNED, Juan del Rosal 16, Madrid, Spain; 10 Geodynamics, Stratigraphy and Palaeontology Department, Complutense University of Madrid, José Antonio Novais 12, 28040 Madrid, Spain; 11 Paleobiology Department, National Natural Sciences Museum—CSIC, José Gutiérrez Abascal 2, 28006 Madrid, Spain; 12 Department of Archaeology and Prehistory, University of Valladolid, Valladolid, Spain; 13 Department of Prehistory and Archaeology, Universidad Nacional de Educación a Distancia, UNED, Paseo Senda del Rey, Madrid, Spain; 14 IPHES, University Rovira I Virgili, Tarragona, Spain; 15 Department of Cartographic and Terrain Engineering, Superior Polytechnic School of Ávila, University of Salamanca, Salamanca, Spain; 16 Department of Topographic and Cartography Engineering, Higher Technical School of Engineers in Topography, Geodesy and Cartography, Universidad Politécnica de Madrid, Mercator 2, 28031 Madrid, Spain; 17 Department of Anthropology, Rice University, 6100 Main St., Houston, TX 77005-1827 USA; 18 Computational Archaeology (CoDArchLab) Institute of Archaeology, University of Cologne, Albertus-Magnus-Platz D-50923, Cologne, Germany

**Keywords:** Archaeology, Biological anthropology

## Abstract

Humans are unique in their diet, physiology and socio-reproductive behavior compared to other primates. They are also unique in the ubiquitous adaptation to all biomes and habitats. From an evolutionary perspective, these trends seem to have started about two million years ago, coinciding with the emergence of encephalization, the reduction of the dental apparatus, the adoption of a fully terrestrial lifestyle, resulting in the emergence of the modern anatomical bauplan, the focalization of certain activities in the landscape, the use of stone tools, and the exit from Africa. It is in this period that clear taphonomic evidence of a switch in diet with respect to Pliocene hominins occurred, with the adoption of carnivory. Until now, the degree of carnivorism in early humans remained controversial. A persistent hypothesis is that hominins acquired meat irregularly (potentially as fallback food) and opportunistically through klepto-foraging. Here, we test this hypothesis and show, in contrast, that the butchery practices of early Pleistocene hominins (unveiled through systematic study of the patterning and intensity of cut marks on their prey) could not have resulted from having frequent secondary access to carcasses. We provide evidence of hominin primary access to animal resources and emphasize the role that meat played in their diets, their ecology and their anatomical evolution, ultimately resulting in the ecologically unrestricted terrestrial adaptation of our species. This has major implications to the evolution of human physiology and potentially for the evolution of the human brain.

## Introduction

Carnivory in early *Homo* is a contentious academic issue. Articulated in the hunting-scavenging debate, archaeologists have produced for decades heuristically-heterogeneous interpretations of the same taphonomic evidences from the early Pleistocene archaeofaunal record (see summary of the predominant positions in^1–7^. Based on uncontroversial evidence, early hominin meat acquisition and consumption is coeval with the onset of encephalization in human evolution and its adoption triggered dietary changes that selected for modified postcranial anatomies adapted to different lifestyles from earlier Pliocene hominins^8–15^. The emergence of hominin meat-eating is also coeval with the earliest stone tool use^16,17^. This has also been used to controversially argue that such a dietary change played a crucial role in early hominin cognitive evolution^8,12,18^. Behaviorally, meat acquisition strategies have also been controversial because they impact our perception of how complex human behaviors emerged^1,19–21^.

Early Pleistocene hominins have been argued by some researchers to have been mostly kleptoparasitic when they entered the carnivore guild, both in Africa^22–26^ as well as in their initial colonization of Southern Europe^27–30^. The difference between the hypotheses posited for both regions is that in Africa, it was argued that hominins were passively accessing mostly defleshed carcasses from modern and extinct felids^22,23,31^, whereas in the Iberian peninsula, it has been argued that hominins were having access to large amounts of flesh abandoned mainly by sabertooth felids (namely*, Megantereon* and *Homotherium*), purportedly unable to efficiently deflesh their prey^27–30^. In contrast, it has also been argued that repeated access to bulk flesh in Africa could only have been feasible through either confrontational scavenging^32,33^, a combination of scavenging of large animals and hunting of smaller game^4^, or a combination of both strategies with predominance of hunting^1,2,34,35^.

Experimentally-replicated models of bone surface modifications (BSM) on early Pleistocene archaeofaunas, when BSM are considered simultaneously in a multivariate format, statistically suggest that anthropogenic sites from this period are most similar to experiments replicating primary access to fleshed carcasses^36–40^. Among BSM, cut marks imparted on fossil bones are the most important traces of hominin carcass butchery. Although some gross anatomical patterning in cut mark distribution was previously shown^35,41^, cut mark analyses have remained under scrutiny because they have been argued to be subjected to equifinal scenarios and to stochasticity^22,42–44^. Equifinality of cut mark patterns has been shown to be an artifact of method^5,45^. Regarding the widely held assumption that cut marks are random accidents on bones, it has also been shown that butchery creates channeling that is widely constrictive as to where cut marks appear clustered and scattered when dealing with complete or incomplete carcasses^46^. Therefore, cut mark anatomical distribution is largely reflective of butchery behaviors and these depend on whether carcasses were acquired completely fleshed or partially or substantially consumed by other carnivore commensals. This enables approaching hominin strategies of carcass acquisition depending on how complete carcasses were upon their exploitation by these agents. Here, kleptoparasitism is understood as a strategy where the bulk of food is obtained through stealing it from other predators^47,48^. This hypothesis will be tested in the present study for early Pleistocene hominins.

Objective analytical procedures to understand BSM anatomical patterning, based on 3D distribution of marks on bones, have recently been developed^46^. This makes classification of any given archaeofaunal assemblage within a set of referential frameworks an objective process. Here, we expand this methodology in conjunction with the use of Deep Learning (DL) analytical methods and state-of-the-art time series classification algorithms to understand if early *Homo* was having primary access to bulk flesh or secondary access to carcasses initially consumed by other predators. The former would probably have triggered important behavioral novelties in human evolution, such as intentional food-sharing and eusociality. The latter (especially if carcasses were mostly defleshed) would most likely not have required such behavioral and socio-reproductive modifications compared to the socio-reproductive strategies of humans´ closest primate relatives.

**Figure 1:**
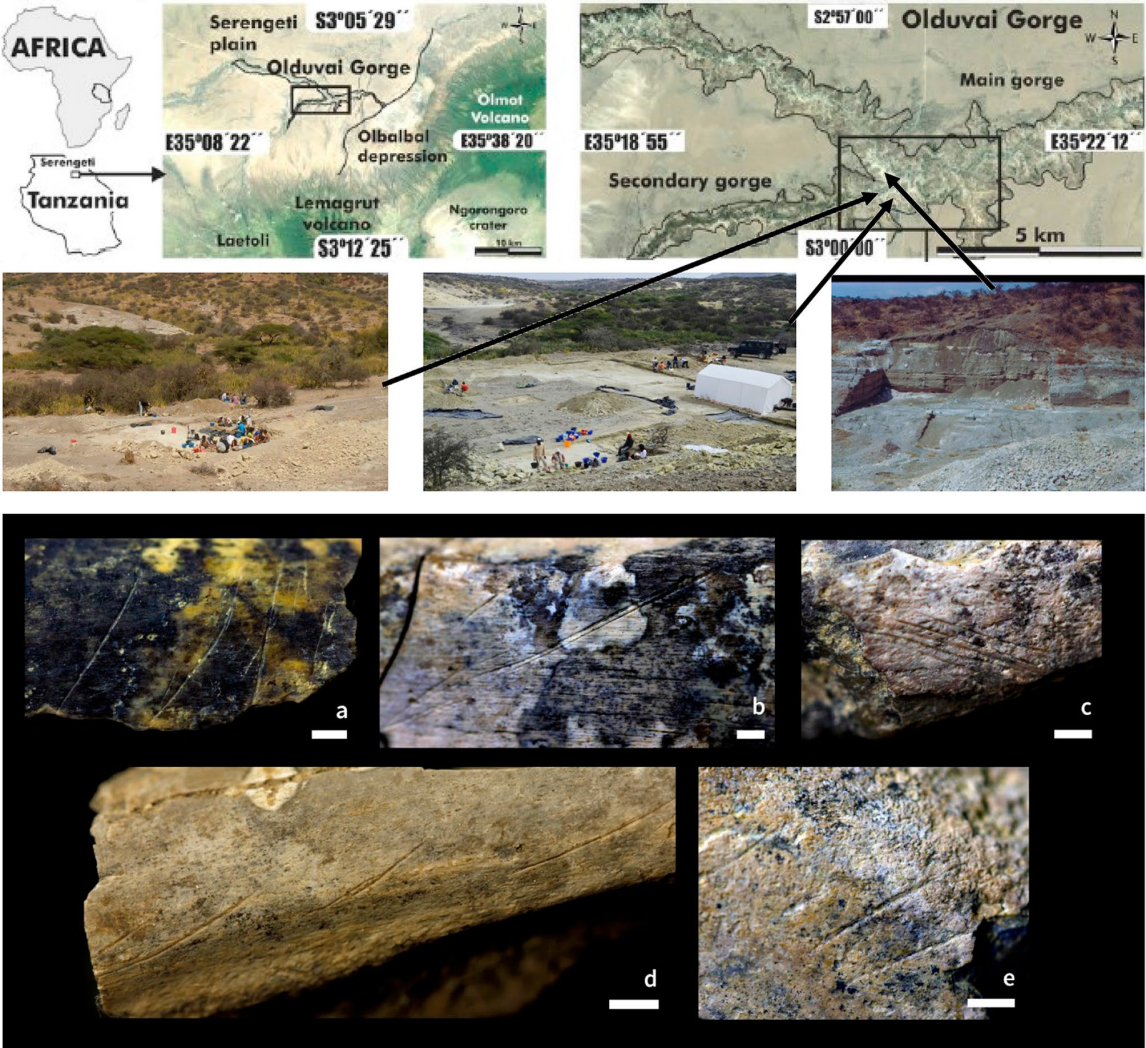
(**Upper half**) location of Olduvai Gorge in Tanzania and images of the PTK (left) DS (center) and FLK Zinj (left) sites during excavation. (**Lower half**) several examples of cut marked humerus and radius shafts from DS. (**a**) Cranio-lateral side of a radius midshaft fragment, (**b**) caudal side of a radius midshaft fragment, (**c**) medial side of a humerus midshaft fragment, (**d**) lateral side of a radius proximal shaft fragment, (**e**) medial side of a humerus midshaft fragment. The map in A was made using Google Earth Pro 7.3.3 (https://www.google.com/earth/versions/).

In order to study early Pleistocene hominin carcass acquisition strategies, we analyze cut mark anatomical distribution patterns in the anthropogenic site of FLK Zinj and in two of the most recently discovered and best preserved early Pleistocene anthropogenic sites in Africa: DS and PTK, (Bed I, Olduvai Gorge, Tanzania) (Fig. 1), dated to 1.84 Ma (see description in Methods). We compare these assemblages to wide-variance experimental sets reproducing primary and secondary access to carcasses in various stages of defleshing (including carcasses obtained at lion kills). Carcasses in these experiments were butchered with the aid of stone tools in both scenarios. Deer and sheep carcasses were used for the butchery experiments. Warthog, zebra and wildebeest carcasses were obtained from lion kills and were also processed with stone tools. The analogs used are composed of two augmented datasets; one using within-sample combinations (combined baseline sample) and another one creating new cases from a latent space using Generative Adversarial Networks (GAN). The goal of these two data sets was to increase within-sample variance so that opposing scenarios could be more reliably identified when contrasted with archaeological samples. Two types of approaches were adopted: one targeted cut mark intensity (frequency of cut marks and anatomical distribution, using raw data), and the other one focused on anatomical patterning (using only relative anatomical distribution of cut marks). These datasets were analyzed using time-series longitudinal classification machine learning algorithms. Six different algorithms were used in order to analyze convergence or not of results. Additionally, ensemble learning was used for classification. This was meant to reinforce the classification obtained by the time-series algorithms. The combination of the quantitative methods used in both cases provide unambiguous evidence of the carnivorous behavior of our ancestors (For detailed description of the methods see Supplementary Information).

## Results

### The combined baseline sample

#### Time Series algorithms and ensemble learning

Four of the six algorithms applied on the raw data yielded accurate (100%) classifications of the testing sets of the primary and secondary access experimental subsamples (Table 1), with others following closely (> 80%). Three algorithms applied on the relative experimental data also produced perfect classification (100%) of the testing set. In both cases, the feature extraction classifiers excelled at classifying the testing sets; especially the WEASEL algorithms (Table 1). Markov Transition Fields also achieved complete classification (using a Random Forest classifier). The high accuracy of most algorithms and the convergence of several of them in classifying correctly all the testing set produced a reliable multi-testing framework within which the subsequent classification of the Olduvai sites was warranted.

**Table 1:** Time Series algorithms used on the combined experimental data set and on the archaeological samples and their results on the raw data set and on the relative data set.

Time Series Classifiers	Configuration	Combined augmented sample		DS	PTK	FLK Zinj
		Raw data	Relative data			
1-KNN (K-nearest neighbour)	metric = 'DTW' (Dynamic Time Warping)	0.961	1.00	P/P (1.00/1.00)	P/P (1.00/1.00)	P/P (1.00/1.00)
2-NN BOSS VS (one nearest neighbour bag-of-SFA-symbols vector space)	word_size = 2–4, n_bins = 2	1.00	0.846	P/P	P/P	P/P
3-SAX-VSM (Symbolic Aggregate ApproXimation and Vector space Model)	word_size = 6, n_bins = 2, strategy = 'normal'	0.807	0.882	P/P	P/P	P/P
4-BOSS (Bag Of Symbolic-Fourier-Approximation Symbols)	word_size = 4, window_size = 119 classifiers = KNN, LSVM	1.00	0.92	P/P	P/S	P/S
5-WEASEL (Word ExtrAction for time SEries cLassification)	word_size = 4, window_sizes = (5, 110/80) Classifier = Logistic Regression (solver = 'liblinear')	1.00	1.00	P/P	P/P	P/P
6-MTF (Markov Transition Field)	Classifiers = Logistic Regression (solver = 'liblinear') Random Forest	1.00	1.00	P/P (0.91/0.93)	P/P (0.92/0.87)	P/P (0.91/0.91)

Results for experiments show the accuracy in the classification of the testing set. Results for the archaeological assemblages show classification according to experimental set (P, primary access; S, secondary access) and probabilities according to reference data set: first (raw data set), second (relative data set). Only KNN and Markov Fields provide probability of classification for the archaeological assemblages. Classification for archaeological data sets is first for raw data (P or S) and then for relative data (P or S).

Once the raw data set was tested, the algorithms were used to test the relative data set, which resulted from the percentage transformation of the raw data set. This transformation sought to analyze anatomical patterning irrespective of the number of cut marks documented. The total number of marks in each assemblage was modified so that each bin in the series contained the percentage value of marks with respect to the complete assemblage. The raw data set represents the patterning and intensity (i.e., frequency of clustering marks) analog, and the relative data set represents just the anatomical patterning analog. Thus, assemblages with widely different numbers of cut marks, but similar anatomical location could be identified. Intensity is so dependent on the interplay of so many variables (with a high degree of randomization), that it can be misleading. Here, we will emphasize the relative anatomical distribution of cut marks, because it is more directly indicative of butchery behaviors and access type to carcasses. Using this relative data set, the Time Series algorithms also succeeded in differentiating bone assemblages modeling primary and secondary access to carcasses. When comparing the archaeological assemblages to the experimental analogs, most of them classify FLK Zinj, DS and PTK as “primary access”, both in cut mark intensity (raw data) as well as anatomical patterning (relative data). The Markov Transition Field algorithm classifieds them as “primary” with a high probability: 0.91–0.91–0.92 on raw data respectively, and 0.91–0.93–0.87 using the relative data set.

**Figure 2:**
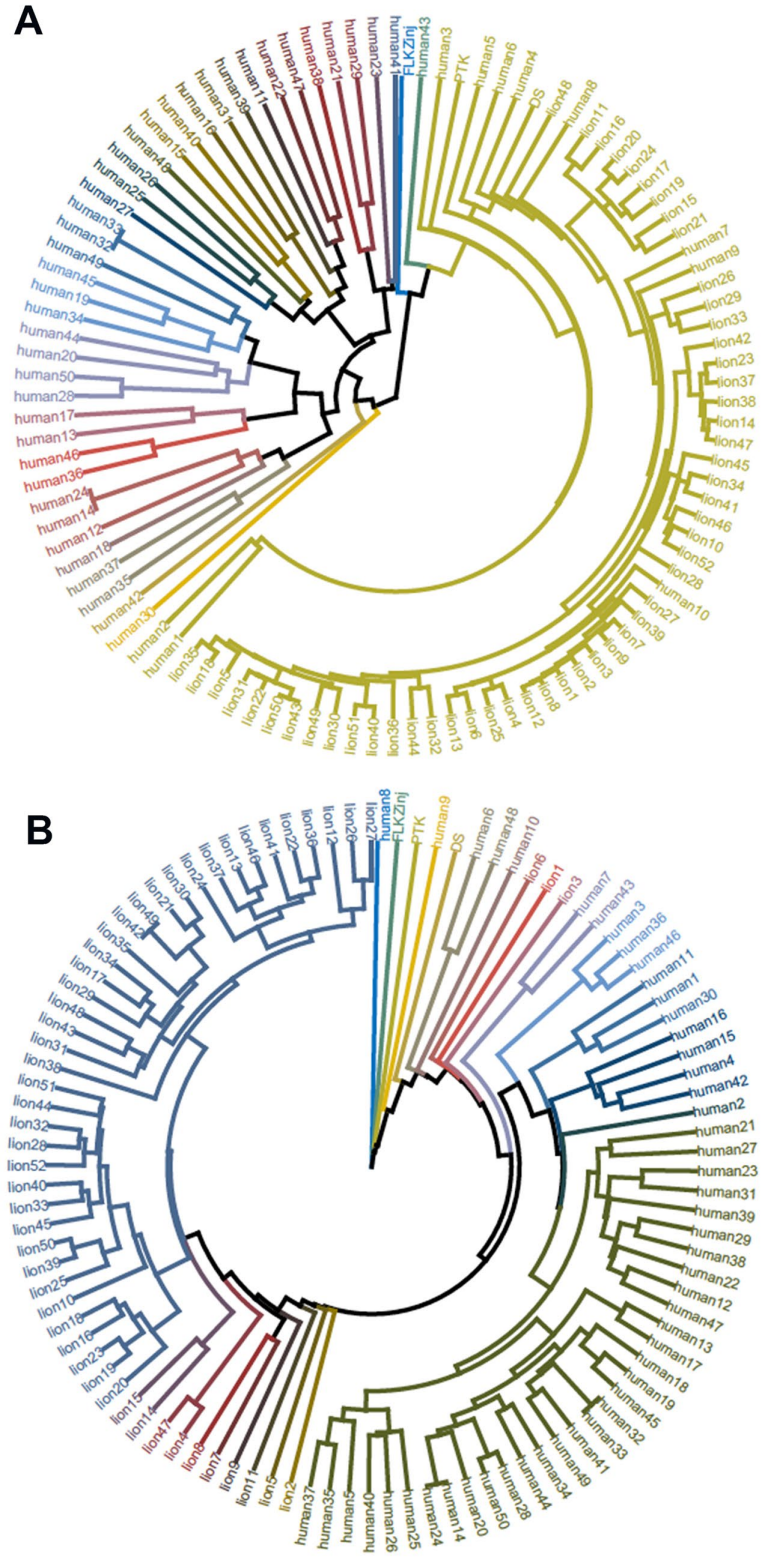
Circular dendrograms showing association of samples in each experimental data set (using the baseline sample), based on similarity/dissimilarity distances (average method). (**A**) Cluster based on raw data. (**B**) Cluster based on the relative data set. Each color shows different groups of samples selected by the algorithm (25 groups). Notice when using raw data (**A**) how all the lion samples are clustered within the same group, whereas the human butchery sample displays a far wider variety and comprises most of the remaining cluster groups. This indicates much more randomness in cut mark intensity when humans butcher complete carcasses and far less so when they process lion-consumed carcasses, because the limited available resources restrict the anatomical portions where tools are used. When using relative data (**B**), the pattern is reversed. Notice how the human samples, which were far more variable in number of cut marks (intensity), reflect much more homogeneous patterning regarding their anatomical distribution. In this case, the lion samples are much more variable, because they reflect higher anatomical variability in the surviving scraps of flesh after lion consumption. Anatomical patterning, thus, is an information rich way of understanding how much meat hominins extracted from carcasses.

This clear divergent classification of both experimental assemblages is also reflected in distance cladograms of each sample per group (Fig. 2). Both the complete carcass butchery and the Tarangire lion butchery sets appear clearly separated in most of the individual samples, which shows that the anatomical patterning of cut marks as well as the frequencies of cut marks in both experimental scenarios are different. Despite the channeling (i.e., constricted occurrence of marks) documented in the clustering of cut marks in certain *loci* due to butchery ergonomics and muscle insertions shared by fleshed and partially or totally defleshed carcasses^46^, resulting in a portion of mark clustering in similar areas, bulk defleshing impacts additionally in areas that show no flesh scraps when carcasses are obtained opportunistically from felid kills. Also, bulk defleshing generates more cut marks as a result of anatomically more extensive meat removal. This creates a clear diagnosis of primary and secondary access scenarios against which the archaeological record can be tested.

Ensemble learning (EL) applied to the relative data sets yielded similar results. A stacked model was used, based on the use of a single-layer of DL (neural networks with multiple hidden layers) base learners and a DL meta-learner. This resulted in perfect classification (100%) of the testing set. Four base learners reached a classification of 100% and one of 97% of the experimental samples (F-1 score = 1.00). DS, FLK Zinj and PTK were classified as “primary access”. Additionally, a majority vote ensemble model was used with the same base learner composition (testing set classified with 100% of accuracy, F1-score = 1.00), and DS, FLK Zinj and PTK were classified as “primary” with a probability of 99%, 94% and 97.9% respectively.

### The GAN-augmented sample

The data augmentation carried out with the use of GAN resulted in 150 new cases (i.e., artificial carcasses) for each experimental group (primary and secondary access). Time Series algorithms classified DS and PTK mostly as “primary”, but with a decrease in accuracy of the testing sets and of the classification probabilities of the archaeological sets, which indicated a wider diversity and variance within the augmented data compared to the baseline data set (Table 2). Despite this artificial increase in within-sample variability, the algorithms reinforced the classification of FLK ZInj, DS and PTK as similar to the experimental data set replicating butchery of complete carcasses. In this case, some algorithms showed that at least one of the sites (PTK) showed similarities with the “secondary” data set when considering the raw data. In contrast, when using anatomical patterning, most algorithms coincide in classifying PTK as “primary”. This stresses the importance of patterning (i.e., where marks occur) over intensity (i.e., how many marks are documented) in determining butchery behaviors, given the higher stochasticity of the former.

**Table 2:** Time Series algorithms used on the GAN-augmented experimental data set and on the archaeological samples and their results on the raw data set and on the relative data set.

Time Series Classifiers	Configuration of parameters	Combined augmented sample		DS	PTK	FLK Zinj
		Raw data	Relative data			
1-KNN (K-nearest neighbour)	metric = 'DTW' (Dynamic Time Warping)	0.86	0.70	P/P (1.00–1.00)	P/P (1.00–1.00)	P/P (1.00–1.00)
2-NN BOSS VS (one nearest neighbour bag-of-SFA-symbols vector space)	word_size = 2–4, n_bins = 4	0.82	0.71	P/P	S/P	P/P
3-SAX-VSM (Symbolic Aggregate ApproXimation and Vector space Model)	word_size = 6, n_bins = 2, strategy = 'normal'	N.C.*	0.63	-/P	-/S	-/S
4-BOSS (Bag Of Symbolic-Fourier-Approximation Symbols)	word_size = 4, window_size = 100 classifiers = KNN, LSVM	0.69	0.61	P/P	S/P	P/P
5-WEASEL (Word ExtrAction for time SEries cLassification)	word_size = 7,4 window_sizes = (5/10, 110/80) Classifier = Logistic Regression (solver = 'liblinear')	0.90	0.75	P/P	P/P	P/P
6-MTF (Markov Transition Field)	Classifiers = Logistic Regression (solver = 'liblinear') Random Forest image_size = 0.1, n_bins = 5–7	0.79	0.62	P/P (0.63/0.56)	P/P (0.53/0.52)	P/P (0.55/0.51)

Results for experiments show the accuracy in the classification of the testing set. Results for the archaeological assemblages show classification according to experimental set (P, primary access; S, secondary access) and probabilities according to reference data set: first (raw data set), second (relative data set). Only KNN and Markov Fields provide probability of classification for the archaeological assemblages. Classification for archaeological data sets is first for raw data (P or S) and then for relative data (P or S).

*No convergence.

When applied to the GAN-augmented relative data set, the ensemble learning models yielded similar results to the combined baseline sample (see above). The stacked DL model yielded an accuracy of 71% (F1 = 0.70) of correct classifications on the testing set. This was substantially lower than the original combined carcass sample which was the base for GAN data augmentation, suggesting that the GAN augmentation had increased variance. Despite this, both PTK and DS were classified as “primary” assemblages. The majority voting ensemble model yielded, in contrast, complete accuracy in the classification of the testing set (100% of accuracy, F1-score = 1.00). This EL model classified DS (probability = 0.93), FLK Zinj (probability = 0.79) and PTK (probability = 0.668) as “primary” assemblages.

Despite the occasional discrepancy of some of the algorithms with the classification of PTK, probably impacted by preservation-related issues (bias in long bone portion representation), it must be stressed that all the tests and analyses applied underscore that both, but especially DS and FLK Zinj, can only be confidently classified within the “primary” access experimental data set. This indicates butchery of complete or fairly complete carcasses. Access to complete carcasses in African savannas, if done regularly, can only be achieved through dynamic (ie., predatory) strategies, since most carcasses that die naturally are utterly defleshed in a matter of hours by the intervention of scavengers^31,49,50^. This underscores the carnivore role of hominins in African early Pleistocene savanna biomes and proves that the hypothesis of regular kleptoparasitism among early Pleistocene hominins is false.

## Discussion

The results of the present study show that by the early Pleistocene, hominins were already inserted in the carnivore guild. Their regular access to fleshed carcasses invalidates hypotheses positing the kleptoparasitic role of these ancestors. Like any other predator, hominins would have exploited available opportunities of carcasses found at other carnivore kills^51^; however, we argue that such strategies constituted a minor element in their carcass-acquisition behaviors. Carcasses obtained from felid kills would have registered typical taphonomic signatures (i.e., bone modifications) resulting from prior felid carcass consumption. In the past several years, with an already extensive archaeological record, such traces have been actively sought and only one unambiguous evidence of felid-hominin interaction has been found, at the DS site precisely (under review). Likewise, the first application of DL computer vision methods to the determination of agency in carnivore bone modification in a fossil assemblage showed that, with the aforementioned exception aside, all tooth pits from the DS archaeofaunal assemblage were caused by hyenas (under review). This eliminates the possibility of felids being the primary providers of carcasses for hominins and reinforces the results of the present study showing that the butchery pattern documented is typical of processing of complete (i.e., fully fleshed) carcasses. Carnivore intervention seems mostly restricted to post-depositional damage by durophagous fissipeds. This means that both small and medium-sized animals of up to 350 kg of weight at the two sites analyzed provided large surpluses of flesh for hominin groups.

These results underscore the role of meat in the early Pleistocene hominin diet and in their socio-reproductive behavior. Primary access to the animals present in these sites (also attested by the presence of eventration cut marks on rib fragments) would have required collective participation of, at least, several individuals, under a behavioral framework that implied intentional cooperation and expectation of resource sharing. Meat-eating would have increased diet quality, thus releasing *Homo* dentition from the selective pressure of copying with high strains caused by wide-range vegetarian omnivory. These changes in dentition could also be explained as selection for their use in preprocessing of meat^52,53^. The reduction in tooth size documented for the first time in this phase of human evolution seems to coincide with an increase of the neurocranium, perhaps being coevolving factors. This supports the impact of this dietary change in the morphing of basal metabolic energy allocation and the evolution of the human brain^10,13,18,54^. In addition, it is precisely at this time that we have evidence also of a major skeletal modification in some early *Homo,* with the emergence of *H. erectus*^55,56^. The modern human bauplan (long body, shortened forelimb and expansion of the hindlimb, with barrel-shaped thorax) emerges also at about this time. This probably is a reflection of the anatomical impact that the dietary change towards carnivory produced^8^. If hominins were predators, this would have required substantial behavioral changes resulting in major anatomical modifications compared to the previous Pliocene and the pene-contemporaneous early Pleistocene hominin stocks.

The presence of a nuchal ligament in *H. erectus* is suggestive of the stabilization of the head to the trunk, probably to counter the shock wave effect of the heel strike of the foot during running^57,58^. The fact that other cursor mammal runners have nuchal ligaments suggests that *H. erectus* was also a runner. The co-occurrence of a nuchal ligament with the earliest evidence of long legs in a larger body size (which, although also selected for by walking, are essential for running) supports the interpretation that this hominin taxon engaged in endurance running. Additional evidence for the stability needed during running comes from the long Achilles tendon, the podal plantar arch, the short forefoot and, especially, the enlarged semicircular canals of the ear^57,58^. An expanded *gluteus maximus*, which stabilizes the trunk, as inferred from the larger sacroiliac trough in *H. erectus*, would also have been essential for the proper biomechanical adaptation to running^57,58^. Bramble and Lieberman (2004) showed that the decoupling of head and shoulder muscles, would also have enabled the efficient swinging of the arms while keeping the head fixed and stable during running. Chimpanzees have their heads and shoulders connected by three muscles, which have been modified in modern humans. Only one of these muscles (the trapezius) still connects the shoulder and head in humans. Two independent tubular structures (a long neck and a long waist) also enhanced the trunk rotation during arm swinging to counter the angular momentum caused by the spinal rotation triggered by leg swinging. The first hominin in which all these features, which are clearly fundamental for running, are documented is *H. erectus*. If running was positively selected for the acquisition of animal food, then meat-eating and the evolution of human anatomy were interdependent co-evolving factors.

The predatory role of these hominins could also have left an anatomical imprint in the evolution of their arms. Humans are the most endowed primates to store and release energy at the shoulder needed for efficient throwing. No other primate can do so with as much control and with as much speed, strength and accuracy^59^. Although the capability of *H. erectus* to use their arms efficiently during throwing was questioned by their inferred narrow shoulders, low humeral torsion and anterior position of the scapula^60,61^, it has been shown that *H. erectus* claviculo-humeral ratio falls within the range of variation of modern humans^59^. In addition, no evidence exists in adult *H. erectus* specimens that the low humeral torsion is maintained ontogenetically as documented in the subadult KNMWT15000 individual. Roach and Richmond (2015) have also demonstrated that there is no relationship between clavicle length (i.e., shoulder width) and throwing performance. Biomechanical studies show that fast and strong throwing in modern humans is enabled by three factors: tall and mobile waists, humeral torsion and the laterally-oriented gleno-humeral joint^62^. *H. erectus* is the first hominin in which these three factors can be anatomically documented. However, the humeral torsion of this taxon is lower than modern humans and it is not known how this could have impacted this hominin´s skills at throwing. If the anatomical indications are correct in pointing at the throwing biomechanical efficiency in *H. erectus*, the subsequent inference is that such skill was probably selected for hunting. The evolutionary co-occurrence of these anatomical shifts and the taphonomic evidence in the archaeological record associated with this taxon that hominins were consuming meat provides compelling support to the interpretation that behavior and anatomy co-evolved through positive selection of predation and meat-consumption.

This dependence on meat could also have triggered important changes in early hominin physiology by adapting to a regular consumption of animal protein and fat. There is evidence that modern human physiology, which makes our species highly dependent on regular intake of cobalamine, may also have its origins in the early Pleistocene^63^. Choline, an essential nutrient that plays a crucial role in gene expression (through methylation of its oxidized form, S-adenosylmethionine) and in brain and liver function is also most abundant in meat and animal products, with very few plants containing any substantial amount of it^64,65^. Humans are also more dependent on this essential nutrient than other primates and failure to meet minimum doses leads to serious pathological conditions. Genomic analysis of the trypanosomic *Taenia* (tapeworm) indicates that human-host infection could have started by 1.7 Ma^66,67^, further suggesting that by that time hominins were facultative carnivores. Pathogens (viruses, bacteria, prions) associated with meat consumption limit other primates’ carnivory and suggest that humans evolved meat-specific genes that allowed a more effective buffer against pathogens and meat-related pathologies (e.g., hypercholesterolemia, vascular diseases)^68,69^, as well as hosting a different microbiome more apt for digesting animal fat and protein^70–72^.

The adoption of carnivory for early *Homo* is also relevant from an ecological perspective. Most modern African carnivores can be qualified as anything but occasional kleptoparasites, with the exception of vultures and brown hyenas. The latter survive in moderate to low competitive ecosystems and complement their diet with hunting of small animals and wide-range foraging for insects and plants. Even more kleptoparasitic organisms do not base the bulk of their food acquisition on this strategy, since it is risky and frequently incurs in additional costs compared to self-foraging^73^. Evolutionary Stable Strategy modeling shows that kleptoparasitism is an inefficient strategy unless used as a complement^74^. Opportunistic strategies are ecologically very valuable, because they reflect intensity in resource competition, high competitor biomass and limited herbivore biomass^75^. The higher the degree of trophic competition, the more frequent kleptoparasitic behaviors are and this impacts on predator group size and, indirectly, on prey size^75,76^. Predators affected by opportunistic competitors may buffer this behavior by expanding group size, switching to larger prey and being more successful at defending kills^75^. This shows that kleptoparasitism behaviors are only successful short-term, but cannot be a stable way of acquiring resources^74^; especially in African savannas, where carnivore diversity and high degree of trophic dynamic competition renders it adaptive for only a small number of specialists, such as vultures or brown hyenas. Hominins would have lacked the ability to monitor savanna habitats for miles from the air and their locomotion adaptation (priming endurance over speed) would have prevented them from being efficient scavengers like brown hyenas, which may tread for more than 30–40 km in each foraging bout^77^. Thus, the carnivoran impact of hominins on savanna ecosystems must be interpreted on different grounds.

Given that all the taphonomic evidence from early Pleistocene anthropogenic sites suggest that hominins may have successfully hunted small and medium-sized animals, their ecological impact affected first and foremost the predatory guild. Analytical studies of carnivore functional and evenness richness across the Pliocene and Pleistocene in East Africa show that there has been a loss of functional richness > 99% from the Pliocene until today^78^. Climatic and environmental information does not correlate with carnivore extinctions, especially after 2 Ma^78^. A thorough analysis of the past four million years shows, in contrasts, that there is a strong correlation between carnivore richness decrease and hominin brain expansion, suggesting that an increase in cognitive skills may have enabled hominins to overtake ecological niches occupied by other carnivore taxa during Quaternary^79^. This supports that “anthropogenic influence on biodiversity started millions of years earlier than currently assumed”^79^. If meat-eating allowed for a high-quality diet impacting on higher hominin demographics, hominin expanding densities across landscapes could have pressured several competing carnivores and could have placed the latter at selective disadvantage. Such a demographic increase is also supported by much bigger sizes of Acheulian sites after 1.7 Ma and the conspicuous evidence of megafaunal exploitation (not necessarily through hunting) after this date by hominins, probably suggesting bigger group sizes^36,38,39^, given the ecological correlation between carcass size exploitation and number of carnivore commensals^80–84^. Body-size ecology (including total carnivore mass and pack size) determines targeting specific prey sizes in mammalian predators^80–84^. The increase of hominin body size and anatomical robustness across the early and middle Pleistocene indicates a selection of physical strength, probably either reflecting predatory strategies that required force or an exaptation in this direction.

Although it has recently been argued that no evidence exist for the anthropogenic impact in African Pleistocene mammal faunas^85^, the arguments against it are affected by similar anecdotal assumptions as some arguments in favor of such an impact. The focus on megaherbivores and their higher representation in the past, for example, is biasing and needs to be properly justified. Megaherbivores´ ecological niches are preferentially situated in certain alluvial habitats, which favor their preservation because of the fast sedimentation processes operating in such environments. Most Plio-Pleistocene paleontological and archaeological localities represent portions of alluvial habitats. The greater representation of megaherbivores in the past has certainly been impacted by this and might as well be just a taphonomic artefact affecting our perception of megafaunal paleoecological diversity and biomass. Climatic–based interpretations overlook the bias introduced by taphonomy as much as arguments using taxa richness for ecological transitions. Faith et al.^85^ correctly argument that carnivoran richness and extinction rates can alternatively be reflecting sampling intensity (i.e., number of sites per period). They also counter-argument that grassland expansion could be responsible of such extinctions. However, it has been shown that it is sampling biases (i.e., number of sites and fossiliferous localities per period and collection intensity) that can account for specific taxic diversity and not climatic forcing, especially for the 1.9 Ma period^86^. No climatic explanation currently can be used to account for mammal evolution without taphonomic calibration^86^. Without this, true climatic evolutionary signals cannot be supported. In their own explanation, Faith et al.^85^ detect a decreasing carnivore taxonomic diversity “trend” after 1.9 Ma (and, especially after 1.5 Ma), which they interpret as ecological, while it is also related to the decrease of sampling intensity, given the smaller number of localities and their areal sizes at the end of early Pleistocene in Africa.

Faith et al.^85^ also reject for the sake of their arguments that hominin might be targeting meat-consumption in the Pliocene and early Pleistocene and claim that the early archaeological record merely documents bone marrow exploitation and hominin kleptoparasitic behaviors, against all taphonomic evidence currently available^1,2^. These authors additionally assume (without support) that small-carnivore extinction should precede large carnivore extinction if hominins were a competitive factor. This assumption is also not ecologically justified and also goes against the zooarchaeological evidence showing that starting 2.6 Ma, hominins were targeting medium-sized taxa preferentially, which affects large carnivores far more than small ones. An argument in favor of hominin impact can be found in Faith et al.´s own data, which clearly show an abrupt decline of large carnivore taxa at 1.8 Ma, precisely the age in which our analysis documents strong carnivoran adaptations by hominins consuming prey that is typical of larger carnivores. In addition, it is the megapredators that show a higher degree of specialization on meat, whereas mesopredators tend to be more generalists^87^, rendering the former more susceptible to be affected by competition.

By focusing on all taxa, including viverrids and mustelids, Faith et al. are also missing the point that it is the large carnivore trends that are most important for assessing the impact of hominins on the predatory guild. Additionally, Faith et al. put some faith in the intrinsic association of large carnivores and megaherbivores, when there is a strong record of similarly-sized carnivores and their preferential adaptation to the consumption of medium-sized (100–400 kg) prey, and even smaller animals^88^. No evidence exist that could be used to support taxon-wide association of predator–prey dependence between Homotherium or Pachycrocruta and megaherbivores. In most of the geographic record of Homotherium, for example, the taphonomic evidence of its involvement with megaherbivores is rather limited. It should be emphasized that in Africa, these large carnivores disappeared way before their potential megafaunal prey did.

FLK Zinj, DS and PTK are probably the best preserved of the early Pleistocene anthropogenic sites in Africa spanning the first million years of the archaeological record. They occur as stratigraphically discrete horizontal concentrations of hominin-processed faunal remains. The Kanjera site (Kenya) has also been used as an example of possibly predatory carnivory by early hominins^4^; however, the nature of this deposit presents more potential time-averaging problems that may have impacted on its integrity and resolution. Its structure as a thicker deposit indicates much more time for a potential diversity of biotic and abiotic agents to have intervened and create a palimpsest from which disentangling the hominin part is challenging. As a matter of fact, the reported frequency of anthropogenic BSM is marginal and substantially lower than those reported in experiments and archaeological assemblages where the agency in the accumulation of the assemblage is solely or mostly hominin. It would be interesting to apply the same methods described here to this assemblage.

**Figure 3:**
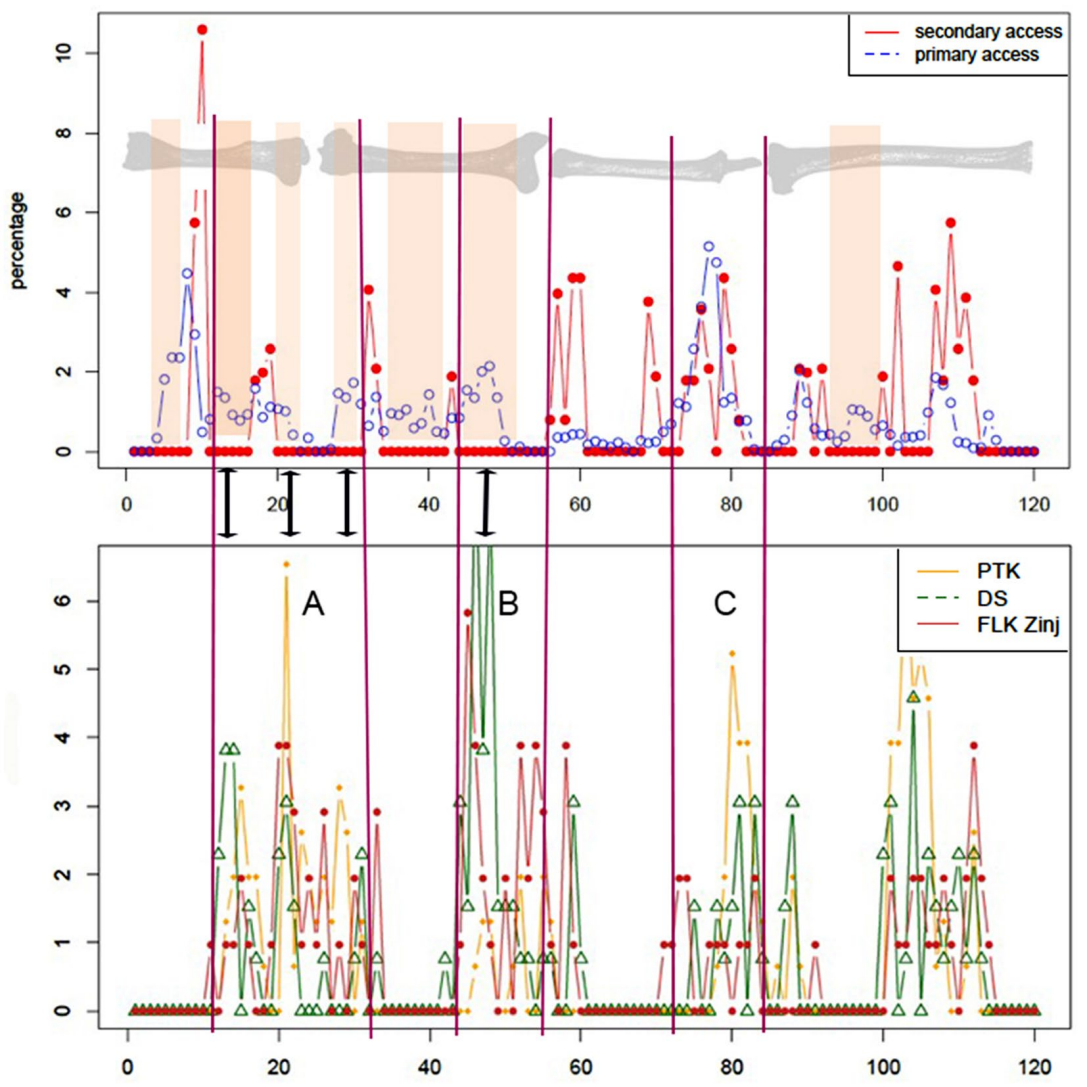
Averaged relative distribution of cut marks in the experimental assemblages (baseline combined sample) replicating primary and secondary access to carcasses (upper) and in the FLK Zinj, DS and PTK archaeological data sets (lower). Colored vertical bands show bone portions where cut marks in “secondary access” experiments are non-existent in the experimental data set, and which would indicate access to fully fleshed carcasses. Notice how cut mark on the DS and PTK stylopodial fragments cluster preferential in these bone portions, suggesting bulk defleshing. Notice also how cut mark patterning in zeugopods are more ambiguous than in stylopods. Three areas of cut mark clustering are documented in the arcghaeological samples conforming to the primary access experiments: (**A**–**C**) A indicates patterning in cut mark clustering on the proximal half of humeri and distal femora. (**B**) indicates patterning on the proximal half of femora. (**C**) Shows that the cut marks pattern documented in the three archaeological samples is virtually identical to the primary access experiments, with clustering on the proximal epiphysis and metadiaphysis and almost no cut marks on the mid-shaft. The position of the tibia is different to the other long bones because it was programmed (scanned) in Ikhnos the opposite way. The X axis show the longitudinal dimensions of the complete series of the four long bones placed sequentially.

The virtually identical patterning in the relative distribution of cut mark clustering on long bones at the three archaeological sites analyzed here (Fig. 3) indicates that: (a) such patterning has a behavioral non-stochastic nature, and b) it clearly indicates a better match with experiments reproducing primary butchery of complete carcasses. The main differences seem to be related to less secondary limb dismembering occurring at the archaeological assemblages. This may have interesting implications on the interpretation of the social behavior of carcass consumption by early hominins, which also seems to have been spatially more restricted than observed among modern human foragers. The relevance of the patterning discovered at the three sites cannot be overemphasized. These are the only taphonomically-supported fully anthropogenic sites in the African early Pleistocene prior to 1.5 Ma^1,2^. It means that if further behavioral variation existed, that must be uncovered with new sites. It also means that the pattern unveiled shows a socio-structural behavior in the adaptation of those early humans. The evidence of primary access and bulk defleshing by hominins at these three sites precedes similar evidence also documented at some sites after 1.5 Ma^2^. Some archaeofaunal assemblages from Olduvai (BK), Peninj (ST4) (Tanzania), and Swartkrans (member 3) (South Africa) have yielded an abundance of taphonomic data indicating that hominins regularly enjoyed early access to prime portions of ungulate carcasses^90–92^.

The data analyzed and discussed here show that if a kleptoparasitic phase existed in human evolution, this must be researched prior to 2 Ma or earlier in the Pliocene^89^. Until more evidence is gathered, we will not know if hominin carnivory was a cladogenetic or an anagenetic event^2^. Nevertheless, its impact on the evolution of human socio-reproductive behaviors, physiology and anatomy is undeniable.

## Supplementary Information

Supplementary Information.

## References

[1] Domínguez-Rodrigo M, Barba R, Egeland CP (2007). Deconstructing Olduvai: a taphonomic study of the Bed I sites.

[2] Domínguez-Rodrigo M, Pickering TR (2021). The meat of the matter: An evolutionary perspective on human carnivory. Azania.

[3] Egeland CP (2012). Model hominid lifeways during the Oldowan. Stone Tools Fossil Bones.

[4] Ferraro JV (2013). Earliest archaeological evidence of persistent hominin carnivory. PLoS ONE.

[5] Domínguez-Rodrigo M (2015). Taphonomy in early African archaeological sites: Questioning some bone surface modification models for inferring fossil hominin and carnivore feeding interactions. J. Afr. Earth. Sci..

[6] Oliver JS, Plummer TW, Hertel F, Bishop LC (2019). Bovid mortality patterns from Kanjera South, Homa Peninsula, Kenya and FLK-Zinj, Olduvai Gorge, Tanzania: Evidence for habitat mediated variability in Oldowan hominin hunting and scavenging behavior. J. Hum. Evol..

[7] Pobiner BL (2020). The zooarchaeology and paleoecology of early hominin scavenging. Evol. Anthropol..

[8] Aiello LC, Wheeler P (1995). The expensive-tissue hypothesis: The brain and the digestive system in human and primate evolution. Curr. Anthropol..

[9] Brains and guts in human evolution (1997). The expensive tissue hypothesis. Braz. J. Genet..

[10] Aiello LC, Bates N, Joffe T (2001). In defense of the expensive tissue hypothesis. Evolutionary anatomy of the primate cerebral cortex.

[11] Aiello LC (2007). Notes on the implications of the expensive tissue hypothesis for human biological and social evolution. Guts Brains.

[12] Konarzewski M, Goncerzewicz A, Knapska E, Dzik J, Rkiewicz TG (2020). Energetic costs of cognitive abilities: Testing the expensive tissue hypothesis. Authorea.

[13] Roebroeks W (2007). Guts and brains: An integrative approach to the hominin record.

[14] Pontzer H (2012). Ecological energetics in early homo. Curr. Anthropol..

[15] Antón SC, Potts R, Aiello LC (2014). Human evolution. Evolution of early Homo: An integrated biological perspective. Science.

[16] de Heinzelin J (1999). Environment and behavior of 2.5-million-year-old Bouri hominids. Science.

[17] Domínguez-Rodrigo M, Pickering TR, Semaw S, Rogers MJ (2005). Cutmarked bones from Pliocene archaeological sites at Gona, Afar, Ethiopia: Implications for the function of the world’s oldest stone tools. J. Hum. Evol..

[18] Navarrete A, van Schaik CP, Isler K (2011). Energetics and the evolution of human brain size. Nature.

[19] Potts R (1988). Early Hominid Activities at Olduvai.

[20] Binford LR (2014). Bones: Ancient Men and Modern Myths.

[21] Isaac G (1978). The food-sharing behavior of protohuman hominids. Sci. Am..

[22] Blumenschine RJ (1995). Percussion marks, tooth marks, and experimental determinations of the timing of hominid and carnivore access to long bones at FLK Zinjanthropus, Olduvai Gorge, Tanzania. J. Hum. Evol..

[23] Capaldo SD (1997). Experimental determinations of carcass processing by Plio-Pleistocene hominids and carnivores at FLK 22 (Zinjanthropus). Olduvai Gorge, Tanzania. J. Hum. Evol..

[24] Selvaggio MM (1994). Carnivore tooth marks and stone tool butchery marks on scavenged bones: Archaeological implications. J. Hum. Evol..

[25] Pante MC, Blumenschine RJ, Capaldo SD, Scott RS (2012). Validation of bone surface modification models for inferring fossil hominin and carnivore feeding interactions, with reapplication to FLK 22, Olduvai Gorge, Tanzania. J. Hum. Evol..

[26] Pobiner BL (2015). New actualistic data on the ecology and energetics of hominin scavenging opportunities. J. Hum. Evol..

[27] Martínez-Navarro B, Fleagle JG, Shea JJ, Grine FE, Baden AL, Leakey RE (2010). Early Pleistocene Faunas of Eurasia and Hominin Dispersals. Out of Africa I: The First Hominin Colonization of Eurasia.

[28] Navarro BM, Palmqvist P (1995). Presence of the African MachairodontMegantereon whitei (Broom, 1937)(Felidae, Carnivora, Mammalia) in the Lower Pleistocene Site of Venta Micena (Orce, Granada, Spain), with some Considerations on the Origin, Evolution and Dispersal of the Genus. J. Archaeol. Sci..

[29] Arribas A, Palmqvist P (1999). On the ecological connection between sabre-tooths and hominids: Faunal dispersal events in the lower pleistocene and a review of the evidence for the first human arrival in Europe. J. Archaeol. Sci..

[30] Espigares MP (2019). The earliest cut marks of Europe: A discussion on hominin subsistence patterns in the Orce sites (Baza basin, SE Spain). Sci. Rep..

[31] Blumenschine RJ (1986). Early hominid scavenging opportunities: implications of carcass availability in the Serengeti and Ngorongoro ecosystems.

[32] Stanford CB, Bunn HT (2001). Meat-Eating and Human Evolution.

[33] Parkinson JA (2018). Revisiting the hunting-versus-scavenging debate at FLK Zinj: A GIS spatial analysis of bone surface modifications produced by hominins and carnivores in the FLK 22 assemblage, Olduvai Gorge Tanzania. Palaeogeogr. Palaeoclimatol. Palaeoecol..

[34] Pickering TR (2013). Rough and Tumble: Aggression, Hunting, and Human Evolution.

[35] Domínguez-Rodrigo M, Pickering TR (2003). Early hominid hunting and scavenging: a zooarcheological review. Evol. Anthropol..

[36] Domínguez-Rodrigo M (2014). On meat eating and human evolution: A taphonomic analysis of BK4b (Upper Bed II, Olduvai Gorge, Tanzania), and its bearing on hominin megafaunal consumption. Quat. Int..

[37] Domínguez-Rodrigo M, Bunn HT, Yravedra J (2014). A critical re-evaluation of bone surface modification models for inferring fossil hominin and carnivore interactions through a multivariate approach: Application to the FLK Zinj archaeofaunal assemblage (Olduvai Gorge, Tanzania). Quat. Int..

[38] Organista E (2015). Did Homo erectus kill a Pelorovis herd at BK (Olduvai Gorge)? A taphonomic study of BK5. Archaeol. Anthropol. Sci..

[39] Organista E (2017). Biotic and abiotic processes affecting the formation of BK Level 4c (Bed II, Olduvai Gorge) and their bearing on hominin behavior at the site. Palaeogeogr. Palaeoclimatol. Palaeoecol..

[40] Dominguez-Rodrigo M, Pickering TR (2017). The meat of the matter: An evolutionary perspective on human carnivory. Azania.

[41] Domínguez-Rodrigo M (2002). Hunting and scavenging by early humans: The state of the debate. J. World Prehist..

[42] Capaldo SD (1998). Methods, marks, and models for inferring hominid and carnivore behavior. J. Hum. Evol..

[43] Lyman RL (1987). Archaeofaunas and butchery studies: A taphonomic perspective. Adv. Archeol. Method Theory.

[44] James EC, Thompson JC (2015). On bad terms: Problems and solutions within zooarchaeological bone surface modification studies. Environ. Archaeol..

[45] Domínguez-Rodrigo M, Hovers E, Braun DR (2009). Are all Oldowan Sites Palimpsests? If so, what can they tell us about Hominid Carnivory?. Interdisciplinary Approaches to the Oldowan.

[46] Pizarro-Monzo M (2021). Do human butchery patterns exist? A study of the interaction of randomness and channelling in the distribution of cut marks on long bones. J. R. Soc. Interface.

[47] Beauchamp G (2013). Social Predation: How Group Living Benefits Predators and Prey.

[48] Nishimura K, Breed MD, Moore J (2010). Kleptoparasitism and cannibalism. Encyclopedia of Animal Behavior.

[49] Domínguez-Rodrigo M (2001). A study of carnivore competition in riparian and open habitats of modern savannas and its implications for hominid behavioral modelling. J. Hum. Evol..

[50] Gidna AO, Kisui B, Mabulla A, Musiba C, Domínguez-Rodrigo M (2014). An ecological neo-taphonomic study of carcass consumption by lions in Tarangire National Park (Tanzania) and its relevance for human evolutionary biology. Quat. Int..

[51] Schaller GB (1972). The Serengeti Lion; a Study of Predator-Prey Relations.

[52] Ungar PS (2012). Dental evidence for the reconstruction of diet in African early Homo. Curr. Anthropol..

[53] Zink KD, Lieberman DE (2016). Impact of meat and Lower Palaeolithic food processing techniques on chewing in humans. Nature.

[54] Fonseca-Azevedo K, Herculano-Houzel S (2012). Metabolic constraint imposes tradeoff between body size and number of brain neurons in human evolution. Proc. Natl. Acad. Sci. U.S.A..

[55] Domínguez-Rodrigo M (2015). Earliest modern human-like hand bone from a new >1.84-million-year-old site at Olduvai in Tanzania. Nat. Commun..

[56] Herries AIR (2020). Contemporaneity of Australopithecus, Paranthropus, and early Homo erectus in South Africa. Science.

[57] Bramble DM, Lieberman DE (2004). Endurance running and the evolution of Homo. Nature.

[58] Lieberman DE, Bramble DM, Raichlen DA, Shea JJ (2009). Brains, Brawn, and the Evolution of Human Endurance Running Capabilities. The First Humans – Origin and Early Evolution of the Genus Homo.

[59] Roach NT, Richmond BG (2015). Clavicle length, throwing performance and the reconstruction of the Homo erectus shoulder. J. Hum. Evol..

[60] Larson SG (2007). Evolutionary transformation of the hominin shoulder. Evol. Anthropol..

[61] Larson SG (2009). Evolution of the Hominin Shoulder: Early Homo. The First Humans—Origin and Early Evolution of the Genus Homo.

[62] Roach NT, Venkadesan M, Rainbow MJ, Lieberman DE (2013). Elastic energy storage in the shoulder and the evolution of high-speed throwing in Homo. Nature.

[63] Domínguez-Rodrigo M (2012). Earliest porotic hyperostosis on a 1.5-million-year-old hominin, Olduvai Gorge, Tanzania. PLoS ONE.

[64] Wiedeman AM (2018). Dietary choline intake: Current state of knowledge across the life cycle. Nutrients.

[65] Derbyshire E (2019). Could we be overlooking a potential choline crisis in the United Kingdom?. BMJ Nutr. Prev. Health.

[66] Hoberg EP (2002). Taenia tapeworms: Their biology, evolution and socioeconomic significance. Microbes Infect..

[67] Hoberg EP (2006). Phylogeny of Taenia: Species definitions and origins of human parasites. Parasitol. Int..

[68] Finch CE, Stanford CB (2004). Meat-adaptive genes and the evolution of slower aging in humans. Q. Rev. Biol..

[69] Finch CE, Stanford CB (2003). Lipoprotein genes and diet in the evolution of human intelligence and longevity. Brain and Longevity.

[70] Zaramela LS (2019). Gut bacteria responding to dietary change encode sialidases that exhibit preference for red meat-associated carbohydrates. Nat. Microbiol..

[71] Lomangino K (2013). Gut bacteria, red meat, and CVD. Clin. Nutr. INSIGHT.

[72] Senghor B, Sokhna C, Ruimy R, Lagier J-C (2018). Gut microbiota diversity according to dietary habits and geographical provenance. Hum. Microb. J..

[73] Flower TP, Child MF, Ridley AR (2013). The ecological economics of kleptoparasitism: Pay-offs from self-foraging versus kleptoparasitism. J. Anim. Ecol..

[74] Broom M, Ruxton GD (2003). Evolutionarily stable kleptoparasitism: Consequences of different prey types. Behav. Ecol..

[75] Carbone C (2005). Feeding success of African wild dogs (Lycaon pictus) in the Serengeti: The effects of group size and kleptoparasitism. J. Zool..

[76] Vucetich JA, Peterson RO, Waite TA (2004). Raven scavenging favours group foraging in wolves. Anim. Behav..

[77] Mills MGL (2003). Kalahari Hyenas: Comparative Behavioral Ecology of Two Species.

[78] Werdelin L, Lewis ME (2013). Temporal change in functional richness and evenness in the eastern African plio-pleistocene carnivoran guild. PLoS ONE.

[79] Faurby S, Silvestro D, Werdelin L, Antonelli A (2020). Brain expansion in early hominins predicts carnivore extinctions in East Africa. Ecol. Lett..

[80] De Cuyper A (2019). Predator size and prey size-gut capacity ratios determine kill frequency and carcass production in terrestrial carnivorous mammals. Oikos.

[81] Vézina AF (1985). Empirical relationships between predator and prey size among terrestrial vertebrate predators. Oecologia.

[82] Tsai C, Hsieh C, Nakazawa T (2016). Predator–prey mass ratio revisited: Does preference of relative prey body size depend on individual predator size?. Funct. Ecol..

[83] Portalier SMJ, Fussmann GF, Loreau M, Cherif M (2019). The mechanics of predator-prey interactions: First principles of physics predict predator-prey size ratios. Funct. Ecol..

[84] Loveridge AJ (2009). Changes in home range size of African lions in relation to pride size and prey biomass in a semi-arid savanna. Ecography.

[85] Faith T, Rowan J, Du A, Barr A (2020). The uncertain case for human-driven extinctions prior to Homo sapiens. Quatern. Res..

[86] Maxwell SJ, Hopley P, Upchurch P, Soligo C (2018). Sporadic sampling, not climatic forcing, drives observed early hominin diversity. Proc. Natl. Acad. Sci..

[87] Viranta S (2003). Geographic and temporal ranges of middle and late miocene carnivores. J. Mammal..

[88] Domínguez-Rodrigo, M., Egeland, C. P., Cobo, L., Baquedano, E., Hulbert, R. Sabertooth carcass consumption behavior and the dynamics of Pleistocene large carnivoran guilds. Quaternary Science Review (under review). 10.1038/s41598-022-09480-7 PMC9061710 35501323

[89] Thompson JC, Carvalho S, Marean CW, Alemseged Z (2019). Origins of the human predatory pattern: The transition to large-animal exploitation by early hominins. Curr. Anthropol..

[90] Domínguez-Rodrigo M, Alcalá L, Luque L (2009). Peninj: A Research Project on the Archaeology of Human Origins (1995–2005).

[91] Domínguez-Rodrigo M, Mabulla A, Bunn H, Barba R, Díez-Martín F, Egeland CP, Espílez E, Egeland A, Yravedra J (2009). Unraveling hominin behavior at another anthropogenic site from Olduvai Gorge (Tanzania): New archaeological and taphonomic research at BK, Upper Bed II. J. Hum. Evol..

[92] Pickering TR, Domínguez-Rodrigo M, Egeland CP, Brain CK (2004). New data and ideas on the foraging behaviour of early hominids at Swartkrans Member 3, South Africa. S. Afr. J. Sci..

