# Supplementary material for: Early Pleistocene faunivorous hominins were not kleptoparasitic, and this impacted the evolution of human anatomy and socio-ecology
Source: Sci Rep. 2021 Aug 9;11:16135. doi: 10.1038/s41598-021-94783-4 (PMC8352906; doi:10.1038/s41598-021-94783-4)
Supplement: Supplementary file 1 — Supplementary Information. [file 41598_2021_94783_MOESM1_ESM.docx]

**SUPPLEMENTARY INFORMATION**

**Early Pleistocene faunivorous hominins were not kleptoparasitic, and this impacted the evolution of human anatomy and socio-ecology**

Manuel Domínguez-Rodrigo^1,2^, Enrique Baquedano^1,3^, Elia Organista^4^, Lucía Cobo-Sánchez^1^, Audax Mabulla^5^, Vivek Maskara^6^, Agness Gidna^7^, Marcos Pizarro-Monzo^1^, Julia Aramendi^1^, Ana Belén Galán^8^, Gabriel Cifuentes-Alcobendas^1,2^, Marina Vegara-Riquelme^1,2^, Blanca Jiménez-García^1,9^, Natalia Abellán^1,9^, Rebeca Barba^1^, David Uribelarrea^1,10^, David Martín-Perea^1,11^, Fernando Diez-Martin^12^, José Manuel Maíllo-Fernández^1,13^, Antonio Rodríguez-Hidalgo^1,14^, Lloyd Courtenay^15^, Rocío Mora^15^, Miguel Angel Maté-González^15,16^, Diego Gonzalez-Aguilera^15^

^1^Institute of Evolution in Africa (IDEA), Alcalá University, Covarrubias 36, 28010 Madrid, Spain.

^2^Area of Prehistory (Department History and Philosophy), University of Alcalá, 28801 Alcalá de Henares, Spain.

^3^Regional Archaeological Museum of Madrid, Plaza de las Bernardas s/n, Alcalá de Henares, Spain.

^4^Osteoarchaeological Research Laboratory, Department of Archaeology and Classical Studies, Stockholm University, Wallenberglaboratoriet, SE-106 91, Stockholm,

Sweden.

^5^Department of Archaeology and Heritage Studies, University of Dar es Salaam, P.O. Box 5050, Dar es Salaam, Tanzania.

^6^ The Luminosity Lab, Arizona State University, Tempe, AZ, USA.

^7^Paleontology Unit, National Museum of Tanzania in Dar es Salaam, Robert Shaban St., P.O. Box 511, Dar es Salaam, Tanzania.

^8^CNRS TRACES-UMR5608 Université Toulouse Jean-Jaurès, Maison de la Recherche, 5 allées Antonio Machado, 31058 Toulouse Cedex 9, France.

^9^Artificial Intelligence Department, Universidad Nacional de Educacio´n a Distancia, UNED, Juan del Rosal 16, Madrid, Spain.

^10^Geodynamics, Stratigraphy and Palaeontology Department, Complutense University of Madrid, José Antonio Novais 12, 28040, Madrid, Spain

^11^Paleobiology Department, National Natural Sciences Museum – CSIC, José Gutiérrez Abascal 2, 28006, Madrid, Spain

^12^Department of Archaeology and Prehistory, University of Valladolid, Valladolid, Spain.

^13^Department of Prehistory and Archaeology, Universidad Nacional de Educación a Distancia, UNED, Paseo Senda del Rey, Madrid, Spain

^14^IPHES, University Rovira I Virgili, Tarragona, Spain.

^15^Department of Cartographic and Terrain Engineering, Superior Polytechnic School of Ávila, University of Salamanca, Spain.

^16^Department of Topographic and Cartography Engineering, Higher Technical School of Engineers in Topography, Geodesy and Cartography, Universidad Politécnica de Madrid, Mercator 2, 28031 Madrid, Spain

**Methods and samples**

*The archaeological samples*

FLK Zinj, DS and PTK are located in the junction between the main and secondary branches of Olduvai Gorge in northern Tanzania. All three anthropic assemblages lie on the same paleosurface underneath Tuff IC in Bed I. Their chronology is well constricted by two volcanic tuffs: Tuff IB and Tuff IC, which have been dated by argon-argon (40Ar/39Ar) to 1.848 ± 0.003 Ma and 1.848 ± 0.008 Ma[(*1*)](https://paperpile.com/c/og77al/Dh71K) respectively. The mineralogical composition of the Zinj clay stratum supports the presence of two distinct archaeological levels. Level 22A shows a more hydrologically closed system than level 22B but both sedimentary processes occurred in very low-energy conditions[(*2*)](https://paperpile.com/c/og77al/3dAcT) and the assemblages probably formed in less than one or two years [(*3*)](https://paperpile.com/c/og77al/RdoFm). The highest concentrations of stone tools and fossil bones are preserved in Level 22A at FLK Zinj and PTK and in Level 22B at DS.

The FLK-*Zinjanthropus* archaeofauna has played a critical role in the reconstructions of early hominin behavior during the past fifty years. The site has been studied by several researchers, and a wealth of detailed information exists about its formation and autochthonous nature, the contribution of hominins and carnivores to the as­semblage, the inferred carcass foraging strategies of hominins, the character­istics of the surrounding environment, and the spatial properties of the distri­bution of the remains[(*4*–*15*)](https://paperpile.com/c/og77al/pIoQK+YTMZY+H11Bs+EwD6D+7Qj9N+zvQQ9+T5t3R+t3YeF+U8pfO+jxQbs+EyXgC+cZXXH). Most studies of FLK Zinj have concluded that hominins were actively accumulating carcasses at FLK Zinj, which were probably acquired through primary access (hunting or confrontational scavenging from felids)[(*4*, *7*, *16*)](https://paperpile.com/c/og77al/V66yk+pIoQK+EwD6D).

The bovid MNI of FLK Zinj (based on postcrania) is estimated at 21 carcasses ([(*4*, *7*, *16*)](https://paperpile.com/c/og77al/V66yk+pIoQK+EwD6D)). There are seven small and thirteen medium-size bovids represented, which have been attributed to *Antidorcas recki* (MNI = 7), *Connochaetes* sp., (MNI = 2), *Parmularius altide*ns (MNI = 4), *Kobus sigmoidalis* (MNI = 7) and, additionally, a larger animal represented by *Syncerus sp.* (MNI= 1).

The skeletal part profiles show that all anatomical parts are represented and, although the less dense bones are underrepresented, the axial skeleton makes up about one third (in MNE) of the assemblage. This high presence of axial elements suggests that carcasses may have been transported complete or almost complete by hominins to the site. The well-preserved cortical surfaces of the faunal remains have yielded taphonomic evidence of primary access to carcasses by hominins. Cut mark (20%) (Table 3) and percussion mark (15%) frequencies on appendicular bones indicate that hominins disarticulated, defleshed, and demarrowed the carcasses before carnivores (Domínguez-Rodrigo et al., 2007). After that, carnivores had access post-depositionally to the assemblage and ravaged the bones. Most of the tooth marks are concentrated (14.9%) on the grease-bearing ends of long bones[(*7*)](https://paperpile.com/c/og77al/EwD6D).

At DS, most of the archaeological deposit lay very close to the surface, and intensive excavations by TOPPP (The Olduvai Paleoanthropology and Paleoecology Project) have exposed an area of 554 m^2^, which makes it the biggest open window to the African Early Pleistocene. Level 22B contains the bulk of the archaeofaunal collection and is exceptionally well preserved. This layer has yielded 3458 bone fragments and 1183 lithics, if considering the fraction longer than 20 cm. The assemblage is autochthonous, largely undisturbed, and has preserved its spatial properties, which has enabled conducting a detailed in-site spatial exploration.

There are a minimum of 33 ungulates represented at the site. The majority of the fossil specimens belong to bovids of the genera *Kobus* (MNI = 11), *Parmularius* (MNI = 4), *Megalotragus* (MNI = 2), *Connochaetes* (MNI = 4), *Tragelaphus* (MNI = 2), and *Antidorcas* (MNI = 5). The remaining represented ungulates are *Equus* (MNI = 3), *Hippopotamidae* (MNI = 1) and *Kolpochoerus* (MNI = 1). The DS 22B assemblage presents a strong anthropogenic signature. The distribution of cut marks on long bones indicates that hominins had access to meat resources prior to any other carnivore (Table 3). The predominance of hammerstone breakage patterns suggests that they were also accessing the marrow content of bones. The distribution of cut marks on long bones matches that of experiments that model primary access to fleshed carcasses by hominins. Cut marks appear on 10.2% of the bone specimens. In contrast, tooth mark frequencies are very low (1.9%) and also correspond to experimental scenarios of carnivores having access to hominin hammerstone-broken bones. Felids were not involved in assemblage formation. The prey mortality profiles suggest that the predominant hominin meat acquisition strategy was hunting. DS was probably used as a central place by hominins, and likely formed as the result of the systematic short-distance transport of complete small and several medium-sized carcasses to the site, and the occasional input of partial medium-sized carcasses.

Between 2012 to 2019, twenty-six trenches were opened at PTK generating an area of 185 m2. The mineralogical composition of the Zinj clay stratum supports the presence of two distinct archaeological levels formed at different times with different environmental conditions. The restricted erosion documented at PTK was mostly caused by continuous surface runoff during the wet seasons[(*15*, *17*)](https://paperpile.com/c/og77al/nYyci+cZXXH). The faunal assemblage is dominated mainly by small and medium-size bovids such as *Kobus sigmoidalis* (MNI = 6), *Antidorcas recki* (MNI = 2), *Connochaetes sp.* (MNI = 2), *Parmularius altidens* (MNI = 1), Hippotragini (MNI = 1), *Equus oldowayensis* (MNI = 1), *Hippopotamidae* (MNI = 1) and *Suidae* (MNI = 1). The MNI (20) has been estimated with the postcranial skeleton, and includes nine small carcasses and eleven medium carcasses.

PTK’s level 22a is composed of a dense concentration of stone tools (n = ~ 497) and fossil bones (n = 1265) spatially associated. Overall, the assemblage appears to be well preserved and only minimally affected by post-depositional processes. The faunal assemblage is dominated mainly by small and medium-size bovids as *Antidorcas* sp. and *Kobus sigmoidalis*. The MNI has been estimated at 20 individuals, nine for small carcasses and eleven for medium carcasses. The skeletal part profiles include elements from most of the anatomical areas but the majority are represented by long limb bone shafts meanwhile vertebrae and ribs are under-represented. Appendicular bones are represented by meaty and marrow-rich long limb bones (upper and intermediate limb bones), with high frequencies of butchery (31.5%) (Table S1) and percussion (21.6%) marks on their shaft sections. A scarcity of epiphyseal sections suggests that hyenas may have deleted these portions after hominin butchery. However, the very low incidence of carnivore damage (lower than 6%) suggests a negligible carnivore contribution to the assemblage.

*Table S1. Number of cut-marked specimens from small/medium sized carcasses per meat-bearing long bone element at DS, PTK and FLK Zinj*

| *Element* | *DS-CM/NISP (%)* | *PTK-CM/NISP (%)* | *FLK Zinj-CM/NISP (%)* |
| --- | --- | --- | --- |
| *Humerus* | *14/224 (6.25)* | *14/78 (17.9)* | *25/87 (28.7)* |
| *Radius-ulna* | *21/172 (12.21)* | *3/55 (5.4)* | *27/93 (29)* |
| *Femur* | *13/141 (9.22)* | *8/68 (11.7)* | *11/65 (17)* |
| *Tibia* | *15/187 (8.02)* | *17/9 (21.5)* | *23/130 (17.7)* |

*The experimental samples*

A diverse set of experimental assemblages reproducing butchery of complete carcasses was used. This involved 10 complete carcasses: six deer (*Cervus elaphus*) and four sheep (*Ovis aries*) which were butchered using stone tools. No animal was killed for the purpose of this study. These samples were previously published in analyses of cut mark frequency and anatomical distribution[(*18*, *19*)](https://paperpile.com/c/og77al/Qlk7s+Cd6lE). The cleaning protocols and details of the experiments can be found in the original publications, but are summarized here. The sheep sample was composed of a total of 401 bone fragments (humerus, femora, radio-ulnae and tibiae) belonging to four individuals. They were dismembered by four groups of three butchers, using simple chert and quartzite flakes. After butchery, the bones were cleaned with neutral detergent to remove small scraps of flesh. Then, after bones had dried, cut marks were identified with 10x-20x hand lenses.

The second experimental collection corresponds to six adult deer, acquired in legally organized hunting parties. They were skinned, butchered and disarticulated with three different sets of stone tools, all of them made of flint, corresponding to two carcasses per tool set. The six animals were butchered by the same expert hunter (with 30 years of experience in butchery, using metal tools). Both filleting and disarticulation were made with the animal lying on one side on the floor. The butcher always used stone tools (except for evisceration) and butchery was performed as follows: First, the deer was eviscerated in the field with a metal knife in order to make the carcass lighter for transportation to a secondary butchery spot. This was done carefully, and the only potential marks created during this process may occur on the ventral side of ribs. The animal was then skinned. This activity was carried out by cutting around the proximal metapodial epiphyses, as well as on the medial and lateral sides of tarsals and carpals. The skin was then removed with stone tools aided with pulling actions on the skin. The hunter was assisted by another individual holding the legs of the animal during skinning and disarticulation. When the deer was totally skinned, the next step was to fillet each bone, without disarticulating them. After filleting, disarticulation was carried out from hindlimb to front limb. Femora and humeri were dismembered from pelves and scapulae, respectively. When each deer was completely disarticulated, the bones were buried (always grouped according to individual animal) for six months to allow the soil to clean them naturally. They were then dug up and cleaned only with water and neutral soap to remove adhering sediment.

Additionally, 12 carcasses obtained at lion kills in Tarangire National Park (Tanzania) were also included in the analysis[(*20*)](https://paperpile.com/c/og77al/nNAJ0) (Table S2). After lion consumption, the surviving flesh scraps were also butchered with stone tools. Cleaning and study of the resulting cut marks followed the same protocol as for the complete carcass butchery sample. Bones were inspected under strong light (60W) with hand lenses (10x-20x) and cut marks were introduced into 3D templates of each element using the new Ikhnos software[(*21*)](https://paperpile.com/c/og77al/vEbZ9). Ikhnos is a dynamic 3D geospatial software that allows for the storage, rotation and virtual manipulation of long bones as if they were real elements. Marks can then be digitized on these 3D templates in accordance with their location on the analysed bones. The exact spatial coordinates can be then be obtained and statistically analyzed in an external statistical software environment. For the purpose of this study, the R programming language was used. The resulting mark assemblage(s) using each element can also be displayed in the form of longitudinal series along the A-axis of each long bone. This series (arranged in the same fashion as time series) has been used in an expanded version of the single-bone longitudinal series, by placing all long bones (from upper to lower) sequentially[(*21*)](https://paperpile.com/c/og77al/vEbZ9). Here, we will follow the same approach, and will divide the complete series of the five meat-bearing bones together (humerus, femur, radius-ulna and tibia) into 120 bins, each representing a percentage of the length of each element. Then, cut marks found in each of the bins will be quantified and then compared among equivalent elements from different assemblages. The present study will prime patterning over absolute mark frequency, since the latter is more stochastic and depends on the interplay of several variables, whereas the latter is structurally dependent on the combination of the ergonomics of butchery and flesh availability[(*21*)](https://paperpile.com/c/og77al/vEbZ9).

A recent study of patterning, based on the density (i.e., proportional distribution of marks) and intensity (i.e, their frequency per unit of area) showed that butchery of small and large animals displayed a similar anatomical pattern of cut mark occurrence and clustering[(*21*)](https://paperpile.com/c/og77al/vEbZ9). For this reason, here we include small (sheep) and medium-sized (deer) carcasses in the primary access (i.e., butchery of completely-fleshed carcasses) sample. This diversifies this sample and, thus, increases the diversity and heterogeneity thereof, by expanding its potential overlap with the opposing opportunistic scavenging scenario. We do not seek to minimize inter-sample variance, but to maximize it in order to derive robust classifications by approaching the opposite experimental groups instead of maximizing their separation. The same approach was used with the secondary access (i.e., exploitation of lion kills) sample, which was not limited to medium-sized animals, but also included small carcasses (Table S2). Each carcass was individually entered in the Ikhnos software and counted as one sample unit.

| *Table S2. Tarangire carcasses included in the secondary access subsample for the present study, including the number of lions that consumed them (after* [*Gidna et al. 2014)*](https://paperpile.com/c/XNZjcm/pIyL)*.* | | | |
| --- | --- | --- | --- |
| carcass nº | Habitat | prey | nº lions |
| 1 | Bush | warthog |  |
| 2 | Bush | wildebeest | 7 |
| 3 | Bush | warthog |  |
| 4 | Bush | zebra | 7 |
| 5 | Bush | zebra | 4 |
| 6 | Forest | zebra | 4 |
| 7 | Forest | zebra | 4 |
| 8 | Plain | wildebeest | 7 |
| 9 | Forest | wildebeest | 2 |
| 10 | Forest | wildebeest | 2 |
| 11 | Forest | zebra | 2 |
| 12 | Forest | wildebeest | 7 |

*Analytical methods*

Data augmentation

Given that comparisons among archaeological assemblages show great heterogeneity in frequencies of cut marks, probably due to intrinsic ways in which each archaeofaunal assemblage was butchered, processed and biased by post-depositional processes[(*22*, *23*)](https://paperpile.com/c/og77al/8JHHp+z5tZe), we laid special emphasis on the diversification of variance. For this reason, we implemented a two-stage transformation of the original dataset.

Once the single-carcass sample (baseline sample) was set to 22 carcasses (12 from lion kills and 10 from butchery of complete animals), we augmented it by implementing two alternative strategies. In the first stage, we made random intra-subsample combinations of carcasses; that is, for the primary and secondary access subsamples, we made within-group combinations of 5 carcasses per iteration. This contributed to a double goal. On the one hand, it diversified within-sample variance. Additionally, it provided a better proxy for comparison with the archaeological samples, since both of them were composed of several carcasses each and not just one (as in the experimental baseline samples). We generated 40 random combinations for the Tarangire lion subsample (out of the 3960 possible combinations) and for the complete carcass butchery sample (out of the 1260 possible combinations), so that the combined samples for each scenario amounted to 52 and 50 components for each group respectively. The random generation of these additional assemblages was carried out using random sampling procedures with replacement, similar to bootstrap. This programmed function selected five carcasses at each iteration. This baseline analytical sample was then used for classification by training and testing the time-series algorithms described below with it.

In the second stage, we adopted a different strategy to generate more data and add more variance to the original dataset. We did not use bootstrapping methods, because that leads only to repetition of already existing data (Courtenay and González-Aguilera, 2020). For this reason, we implemented Deep Learning (DL) based methods for the creation of realistic yet synthetic augmented data, using the baseline and combined datasets for both experimental groups (primary and secondary access to carcasses). This expanded the global sample for training as well as provided further diversification of within-sample components and variance. We accomplished this goal by implementing Generative Adversarial Networks (GAN) for tabular data augmentation. Data augmentation has become a major analytical trend[(*24*–*27*)](https://paperpile.com/c/og77al/pwQ6V+YzF1R+OrR2z+g0bCa) since it creates new data that are similar to (but not the same as) the original data. It enables the use of “private” data without legal liabilities by pseudo-replicating it. For science, it provides a very efficient way to amplify sample sizes by enlarging samples and their variances, since the individual cases replicated are unique and under no circumstance two individuals are exactly the same when dealing with multivariate data. This is specially relevant for medical imaging[(*28*–*30*)](https://paperpile.com/c/og77al/6OLLC+ysQ7r+i8Pbl), where samples are always limited. From these fields it has expanded to other analytical disciplines[(*26*, *31*, *32*)](https://paperpile.com/c/og77al/6asE9+4Stvk+OrR2z). GANs were initially developed for image replication and image innovation and these areas are where they became most popular[(*24*, *33*–*38*)](https://paperpile.com/c/og77al/Ltara+pwQ6V+VEtXM+hG41F+H6EqE+8Z9k5+VuPGP).

GANs consist of the competing interplay of two DL neural networks with different functions. One, the generator, creates “fake” data using “real” data. The generator network captures the structure of each individual case of real data and tries to replicate it, without copying it in its entirety. The other network, the discriminator, tries to differentiate between the real data and the “fake” data generated by the generator. Each network creates feedback with the other and both compete to maximize their potential. For the discriminator it is about maximizing differentiation, and for the generator it is about maximizing deception over the discriminator by replicating data as close as possible to the real data. The competition between both neural networks is understood within a Nash equilibrium model (i.e. a zero sum game). The GAN model, thus, requires two neural architectures, two different costs and two different optimizers.

Structure of the GAN model.

For the present analysis, we selected a Conditional Tabular GAN (CTGAN)[(*39*)](https://paperpile.com/c/og77al/jwgT0), which uses labels to train both the discriminator and the generator and is expressly optimized for tabular data. Tabular data augmentation poses unique challenges as it consists of mixed data types, columns with non-gaussian and multimodal distributions and highly imbalanced categorical columns. It is important to find a good representation of the data so that the GAN can learn the distribution without bias. CTGAN uses One-hot encoding for representing the discrete columns. It uses mode-specific normalization instead of a min-max normalization to handle complicated distributions of continuous columns. Finally, a row of data is represented as a concatenation of continuous and discrete columns.

CTGAN uses two fully connected hidden layers in both the discriminator and the generator. Batch-normalization and Relu activation function is used in the generator whereas the discriminator uses the leaky relu function and dropout for each hidden layer. The model uses WGAN loss[(*40*)](https://paperpile.com/c/og77al/lAPef) and Adam optimizer in the training step.

The model uses a conditional generator to ensure that the samples account for minority categories as well. Training-by-sampling technique is used on the output of the conditional generator before feeding it to the discriminator. It discriminator calculates the loss by estimating the distance between the conditional distribution on the synthetic data and the real data.

CTGAN model is evaluated by estimating the Likelihood Fitness and the Machine Learning Efficacy. Likelihood Fitness determines whether the synthetic data follows the same joint distribution as the real data. Machine Learning Efficacy evaluates whether a classification or a regression model trained to predict one column using other columns results in similar accuracies for both synthetic and real data.

For the training of CTGAN, the Wasserstein loss function (WGAN), based on the *Earth-Mover’s distance*[*(40)*](https://paperpile.com/c/og77al/lAPef), was employed. Training was performed for 2000 epochs using the Adam optimization algorithm.

Statistical classification methods

Traditional time series are sequences of data distributed longitudinally along a temporal vector. They are very popular in econometrics, biomedicine, engineering and time-related disciplines. When these sequences are labeled and need to be used for classification, traditional classifiers (including machine learning algorithms) are not well suited because they assume conditional independence among different features (i.e., different components of the sequential series). In time-series sequences, each step is determined by the previous one and this assumption is not tenable. For this reason, specific algorithms have been created to deal with the intrinsic inter-dependence peculiarities of features in time series. All the algorithms that we will use here are adapted to comparisons of longitudinal sequences of the same length. For our analysis, we used the PYTS (Python Package for Time Series Classification) library using Python 3.7. We applied the algorithms using Jupyter Notebooks, with the following dependencies: NumPy 1.19.4, SciPy 1.4.1, Scikit-Learn 0.23.2, Numba 0.52.0 and PYTS version 0.11. PYTS provides preprocessing and utilities tools that prepare data for analysis and contains a large series of algorithms. We used three different types of algorithms. One set was aimed at classifying groups using the time series raw data: NN BOSS VS and SAX-VSM. The other set was based on feature extraction and correlation: BOSS and WEASEL. These types of algorithms are better at capturing correlation of time series features and predicting or classifying label-specific sequences. Finally, we also implemented one imaging time-series algorithms: Markov Transition Fields (MTF). This algorithm transforms time series data into images. These are subsequently classified using DL (including convolutional neural networks). A description of each of the algorithms used is displayed in Table 5. For the feature extraction and correlation algorithms and for the imaging classification algorithms, we stacked (ensemble learning style) additional classifiers. For WEASEL, we used a logistic regression. For MTF, we used logistic regressions as well as random forests (see Table S3).

Table S3. Main TSC (Time Series Classification) algorithms used in the present study.

| **Algorithm** | **Brief description** |
| --- | --- |
| *1-NN BOSS VS (one nearest neighbour bag-of-SFA-symbols vector space)* | *It combines the Symbolic Fourier Transformation with the bag-of-words model resulting in SFA strings for a series of subsequences along the longitudinal data set. It has high accuracy properties and is very robust to noise. The method composes classes using term frequency-inverse document frequency*[*(41)*](https://paperpile.com/c/og77al/rmBMv)*.* |
| *SAX-VSM (Symbolic Aggregate AproXimation and Vector space Model)* | *Based on structural similarity, it uses high-level representation of the longitudinal sequence. It does so through the use of a Bag-of-Pattern approach by means of SAX. This tallies the frequency of similar patterns along a sequence. Data are transformed in a vector space and classification is made using an Euclidean distance to their raw frequencies*[*(42)*](https://paperpile.com/c/og77al/7flAN)*.* |
| *BOSS (Bag-of-Symbols)* | *It extracts subsequences from a series. Local structures are captured with histograms. These offer invariance to distortion and other noises. Structural similarity is found through the use of histograms within a vector space model approach in a similar manner to the bag-of-words structural method.Sliding windows are used and patterns are detected through the use of SAX*[*(43)*](https://paperpile.com/c/og77al/IMxl4)*.* |
| *WEASEL (Word ExtrAction for time SEries cLassification)* | *This method transforms time series into feature vectors across multiple sliding windows. These are subsequently trained through traditional machine learning algorithms. The method uses “aggressive” statistical feature selection, resulting in data dimensionality reduction prior to the application of learners*[*(44)*](https://paperpile.com/c/og77al/Ynddz)*.* |
| *MTF (Markov Transition Field)* | *Based on Tiled Convolutional Neural Networks (TCNN), the method retrieves longitudinal data from the series and transforms them into a Markov Transition Field (MTF). This latter preserves information in the time scale. Data is discretized in the form of quantile bins. The resulting graph is a probability map. MTF creates tiled images that are subsequently analyzed through deep CNN*[*(45)*](https://paperpile.com/c/og77al/Ik8ot)*.* |

For the stage 1 analysis, the experimental sample used consisted of the combined-carcass subsamples (n=52 for the Tarangire lion sample and n=50 for the Complete Carcass Butchery sample). This set was divided into training (70%) and testing (30%) sets. Given that the sample is balanced, no measures of symmetry (e.g., F1-score) were used with the Time Series algorithms to assess balanced accuracy. Before analysis, we compared the 3D data set that we were using to previous data sets that were analyzed merely for cut mark frequencies per bone portion and element and for which 3D data are not available yet[(*20*, *46*)](https://paperpile.com/c/og77al/DTP4N+nNAJ0), and the diversity of locations with cut marks were more restricted in those published samples than in our augmented sample. Thus, data augmentation using a combination of carcasses had successfully reproduced a wider variance than previously documented using single-carcass samples. We were seeking this result, because specifically for the secondary access subsample, we wanted to model different stages of carcass modification following variable distribution of surviving flesh scraps after felid intervention.

Our analysis targeted two goals. One was to compare frequencies of anatomical distribution of cut marks, where more intensive cut marking would reflect different degrees of absolute abundance of cut marks in the anatomy of long bones. If butchery of large amounts of flesh had any reflection in the frequencies of resulting cut marks, we could then compare the contrast in frequency distribution between scenarios modeling primary and secondary access. In this approach, the number of cut marks could be a discriminatory factor between the experimental subsamples. The second goal was not as much concerned with absolute numbers of cut marks (very likely resulting from the interplay of a wide array of contingent circumstances), but with the locations where they appear. The question here was: do different types of access to carcasses show different anatomical patterns in cut mark clustering and distribution? For this purpose, cut marks documented per carcass were displayed in each series bin relative to their global frequency. Carcasses that were marked on the same anatomical locations would then reflect identical patterning, regardless of the amount of cut marks imprinted in each case. To reproduce both scenarios, all the algorithms were first run with the raw cut mark data, and then they were re-run with the relative cut mark data. Given that this anatomical distribution is less dependent on contingency, we further used the relative data sets as reflecting butchery behaviors and to model the two experimentally different types of access to carcasses.

After the classification tests were performed on the combined augmented sample, we proceeded to re-apply them on the GAN-augmented data set. The previous comparison between the raw data and the relative data sets showed that the absolute number of cut marks could bias specific subsample classification; however, when this bias was removed using relative cut mark distribution, distinct cut mark patterning was clearly differentiating the complete carcass butchery sample from the Tarangire lions sample. This also prevented misclassification because of the variable number of potential carcasses involved in any given assemblage. The higher the number the more likely the bias would be in favour of primary access scenarios; the lower the number, the bias would favor secondary access scenarios. For these reasons, we used first the relative data set (reproducing anatomical patterning instead of butchery intensity) as the template for further data augmentation using GANs. With this we generated a dataset targeting anatomical patterning according to behavior (primary or secondary access). Then, we proceeded to generate a second GAN data set targeting anatomical intensity, using then the raw data. Intensity, despite its higher degree of stochasticity, is also important because it can result from intensive removal of flesh scraps (e.g., secondary access to largely defleshed carcasses), or other behavioral variables (e.g., novice or expert butchery of complete animals or more frequent tool-bone contact caused by bulk defleshing).

The GAN network structure, as defined above, was used to generate 150 new samples (evenly divided between both experimental scenarios) for patterning and 150 new samples for intensity. Of the different GAN options available, we selected one, specialized in generating tabular data. We intended to diversify the original sample beyond the baseline sample properties with the goal of approaching the potentially higher variance in the population. This pulls the boundaries of the experimental samples closer and is an excellent proxy for capturing nuances and variability in carcass butchery for both experimental scenarios that were not present in the original samples. Interestingly, the samples thus derived contain probably more variance than if we had expanded the baseline sample with additional real carcasses. The CTGAN that we used was implemented using the “ctgan” Python library. For an abridged and clear introduction to the use of GANs and data augmentation in palaeontology and archaeology see[(*47*)](https://paperpile.com/c/og77al/lt10q).

In both stages of analysis (the combined sample and the GAN-generated expanded sample), the matrices containing the sequential series for the experimental data sets were also analyzed using a hierarchical clustering “average” method. This was performed in R ([www.r-project.org](http://www.r-project.org)).


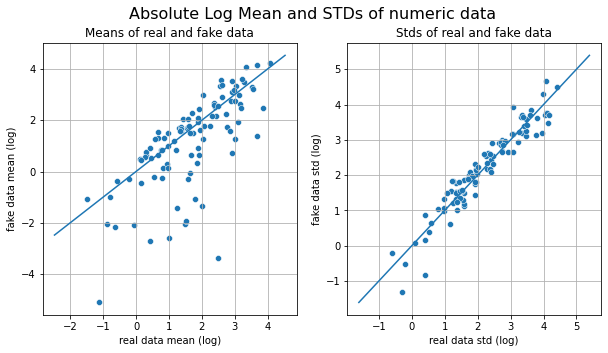


Figure S1. Absolute logarithmic mean and standard deviation of the real data in the relative data set compared to the GAN generated data (i.e., fake data).

Characteristics of the GAN augmented sample.

Prior to the generation of the artificial sample, the original data set displayed lack of data for both experimental groups in 18 bins, corresponding to equal number of small portions of the affected long bones. These were removed and the remaining 102 bins were then fed to the GAN neural network system. These bins were reset after completion of the GAN process, so that 120 bins were then passed to the Time Series algorithms described above. The original relative data was expressed between 0-1 as percentage distribution. GANs do not perform well with data close to zero. Given that GANs perform better with bigger contrasts in data, when using the relative data set, we amplified it 1000 times. This did not affect the proportional distribution of marks among samples or between experimental data sets. We did not follow this protocol when using the raw data. The GAN-augmented relative sample produced an artificial data set that fitted the real set well in its intermediate and upper mean values. More divergence was documented in the lower mean values, which probably constitute the part of the generated sample exhibiting wider variance (Fig. S1). The standard deviation of each of the 102 bins replicated artificially was virtually identical to the original data set, thus showing a similar distribution shape in most of the 120 bins of the sequence (Fig. S1). This can be appreciated in the cumulative sum of data values for both group samples and in the distribution of each of the replicated bins (Fig. S2). The sample kurtosis and shape (skewness) of the real and fake data were very similar and the GAN was as successful as to capture the bimodality of some of the bins subsamples (Fig. S3).


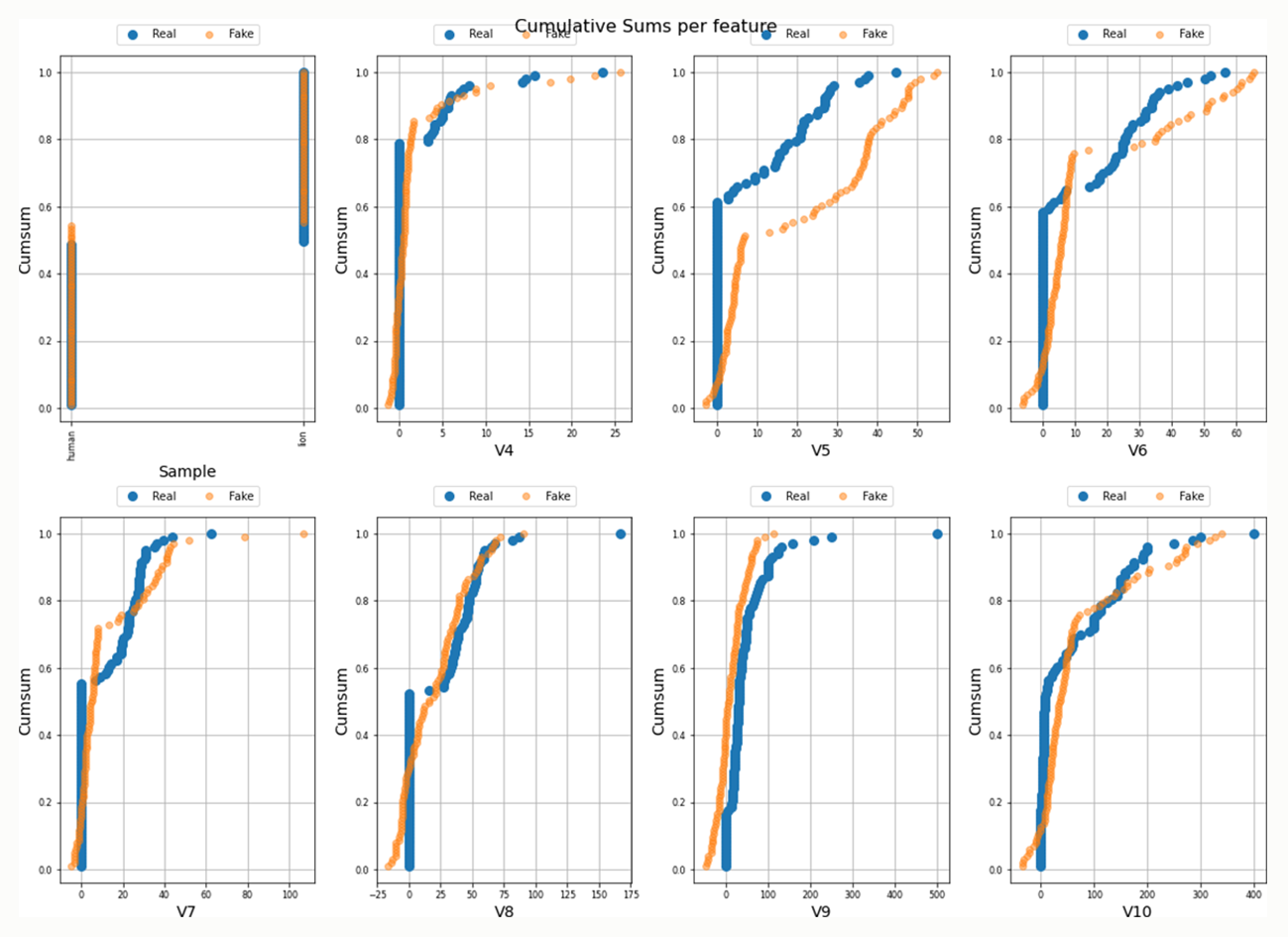


Figure S2. Cumulative sum and shape distribution of the real and the GAN-generated data using the relative data set, and examples of the match between real and fake data in the first seven bins.


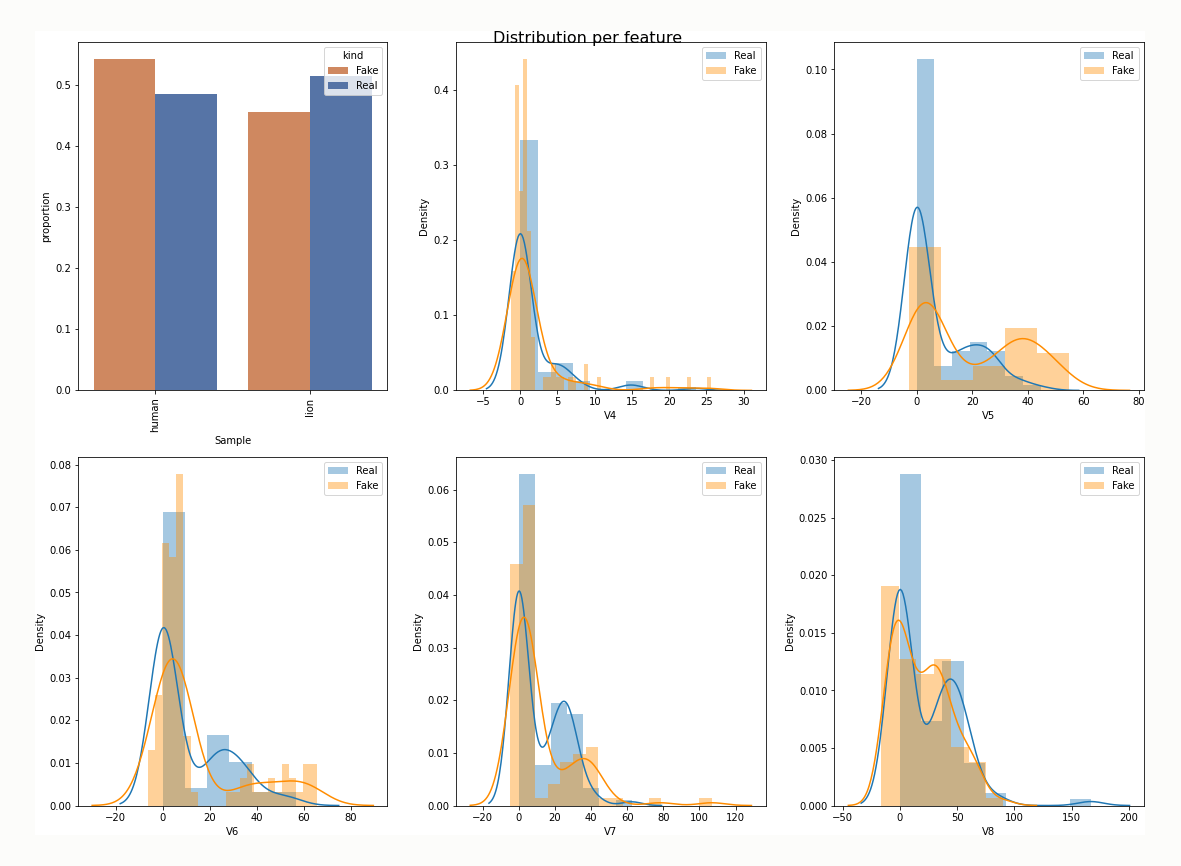


Figure S3. Shape distribution of the real and the GAN-generated data, considering the global distribution of both experimental data sets, and examples of the distributions in the first seven bins of the serial sequence. Real data refers to the relative experimental data sets.


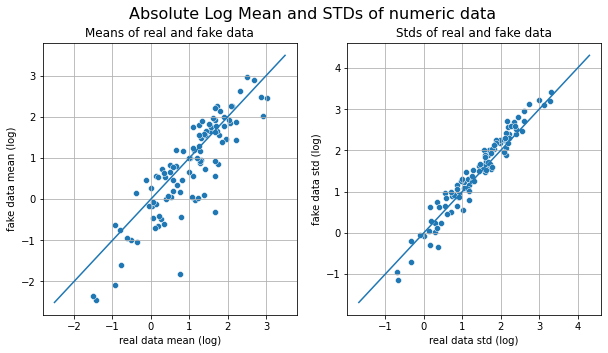


Figure S4. Absolute logarithmic mean and standard deviation of the real data in the absolute raw data set compared to the GAN-generated data (i.e., fake data).

We created an R function to test the variance generated across the 102 GAN bins and compare it to the original data set. It surprisingly showed that the within-sample (i.e., real or artificial data set) variance was very similar. The real data set yielded a combined percentage average of variance of 0.0005 (sd=0.0011) and the artificial data produced an average of 0.0007 (sd=0.0017). The generated data was only slightly more diverse in shape distribution than the real data, but very similar. In contrast, the differences documented at the lower range of the replicated data (Fig. S1) were enough to make the artificial data more diverse and with a higher overlapping trend between experimental scenarios. Two machine learning (ML) classifiers were used to compare both data sets. A support vector machine (SVM) (with radial kernel) and a random forest (RF) (with a maximum of 500 replicates) were used. The parameters used were the default ones from the “caret”´s R library. Data were preprocessed (centered and scaled) and the model was 5-fold cross-validated. This resulted in the testing sets of the real data being classified with an accuracy of 0.933 (SVM) and 0.967 (RF) respectively. In contrast, the artificial data displayed slightly lower accuracy: 0.875 (SVM) and 0.89 (RF). This artificial data set, thus, introduced an element of diversity that was non-existing in the original data set from both scenarios and increased their internal diversity and global variance while showing similar accuracy in its preliminary classification.

Then, when reproducing not just anatomical patterning but also intensity resulting from meat removal, the raw data produced a GAN sample of 300 instances, which showed smaller variation than when using just relative percentages (Fig. S4). The resulting data reflected better the average values of the original data as well as their standard deviations (Fig. S4). The reproduction of data in each bin-variable was in general good (Fig. S5). The generated data, as was the case with the relative data set, also reproduced well the bimodality of some of the variables (Fig. S6). When using the original pre-augmented raw data, 100% of the testing sets were classified accurately (both by SVM and RF). In this case, the GAN augmented data testing set was classified with accuracy ranging between 0.888 (SVM) and 0.90 (RF). The real data produced an average variance of 57.92 (sd= 122.93), while the augmented data yielded an average variance of 78.11 (sd= 148.67). This showed that the augmented data had a slightly bigger spread when considering all bins corresponding to a larger variance. This probably impacted the lower accuracy of the artificial data. In both cases, using percentage distribution of cut marks (patterning) or raw distribution of cut marks (patterning + intensity) resulted in an expanded variance of the original experimental data sets. Interestingly, the preliminary classifiers yield virtually identical classification of the testing sets in both data types. Both were subsequently used for classification of the experimental sets using the Time Series algorithms and ensemble learning (EL).


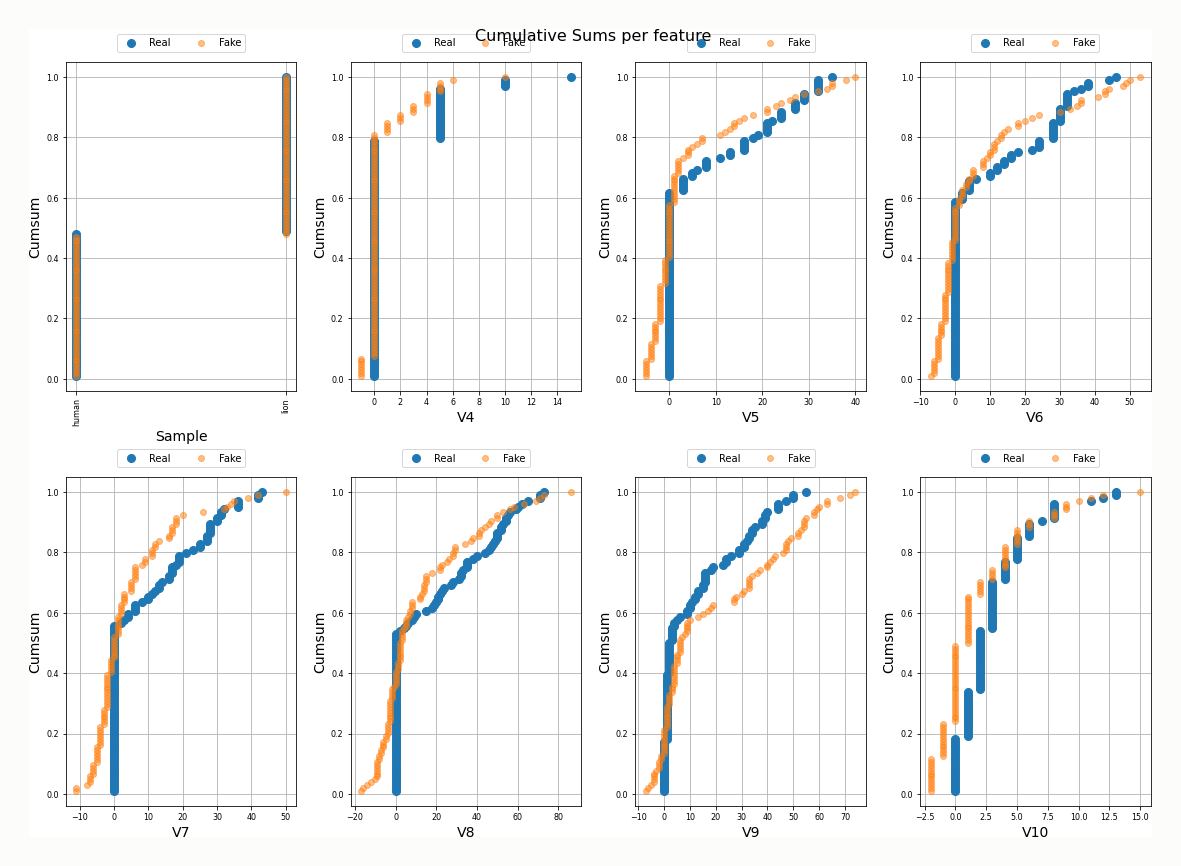


Figure S5. Cumulative sum and shape distribution of the real and the GAN-generated data using the absolute raw data set, and examples of the match between real and fake data in the first seven bins.


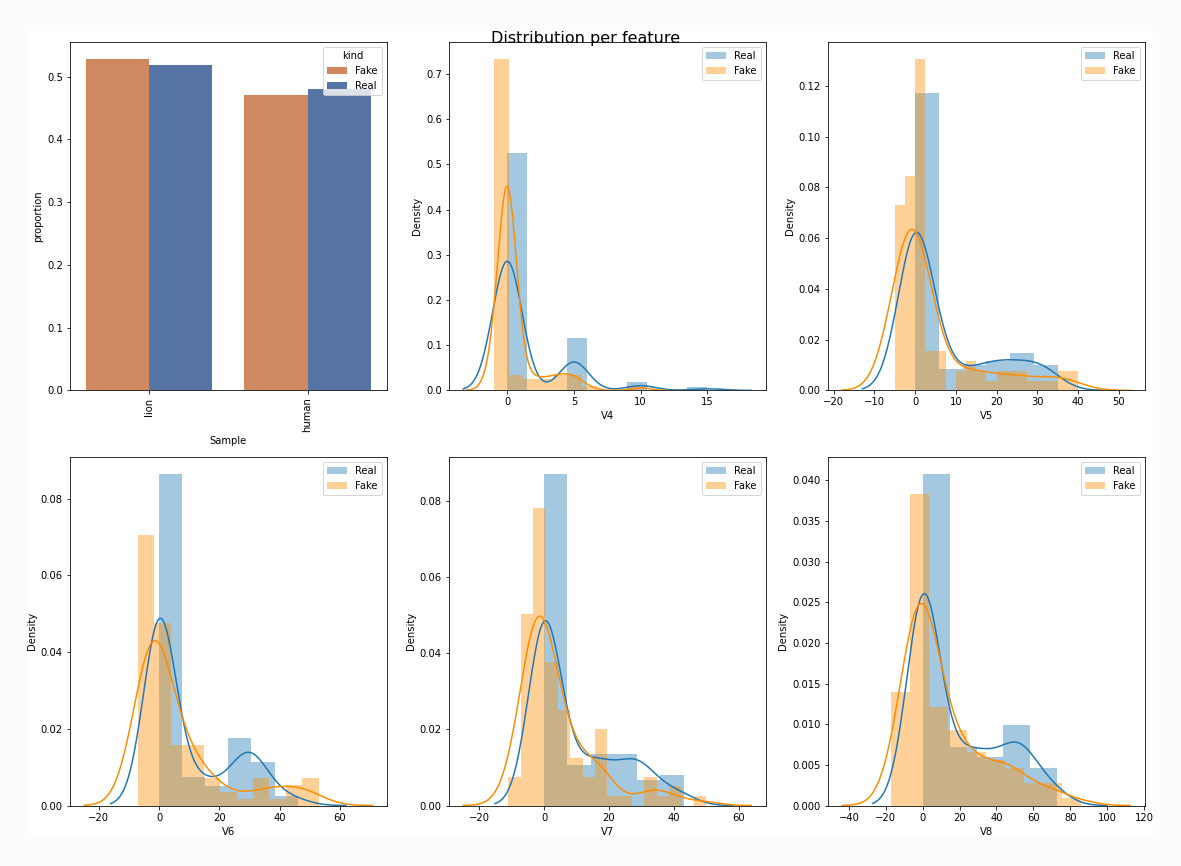


Figure S6. Shape distribution of the real and the GAN-generated data, considering the global distribution of both experimental data sets, and examples of the distributions in the first seven bins of the serial sequence. Real data refers to the absolute raw experimental data sets.

**Ensemble learning**

In addition to the Time Series algorithms, we implemented models from ensemble learning, consisting of the joint use of weights of different learners on training and testing sets to classify the archaeological assemblages. The use of EL constitutes one of the latest advances in machine learning methodology. EL aims at improving the performance of algorithms in regression and classification[(*48*–*50*)](https://paperpile.com/c/og77al/Yh2F2+SzItQ+5xvT6). EL methods consider simultaneously multiple estimators to derive regression and classification decision boundaries. Broadly, there are two kinds of ensemble learning approaches. The first is based on putting together estimators sequentially by decreasing their bias and variance. This is where all the boosting and bagging (bootstrapped aggregating) methods belong. The other approach is based on the diversification of learners, by simultaneously using several types, each of them independently built, and either creating majority voting (classification) or averaging (regression) their results. The former set of EL methods has the advantage of dealing better with the high variability of method adequacy according to the sample characteristics, following Wolpert´s[(*51*, *52*)](https://paperpile.com/c/og77al/hHcON+0Zk6O) “no free lunch theorem”. EL averaging and voting methods allow mixing algorithms as diverse as random forests, neural networks, support vector machines, and even using boosting and bagging algorithms as part of the baseline analysis, such as gradient boosting machines, extra random trees and others.

Two similar EL methods excel at classification: blending and stacking[(*53*)](https://paperpile.com/c/og77al/iN32R). Stacking consists of generating a multiple layer structure of estimators with a hierarchy of base learners at the bottom and one or more meta-learners on top. The outputs of the base models are used by the meta-learners to elaborate the final prediction. Training at the base model level is done with k-fold cross-validation. Blending is a similar approach, since it also uses the base learners as the new-feature estimators. Instead of using a k-fold training data set, blending uses a separate holdout set. Base learners are trained on the training dataset, predictions are made both on the holdout set and the testing sets. The new prediction features are then used to create a meta-model, which in turn is used to make the final predictions.

Here, we used a Stacked model and a Majority Voting model based on a Deep Learning (DL) group of base learners and meta-learner showing the composition and classification results on the relative data set described in Table S4.

*Table S4. Neural network architectures used for the EL stacked model and accuracies on their application on the testing sets of the data-augmented samples.*

| Model | Hidden layer sizes | Batch size and max_iter | Meta-learner hidden layer size | Accuracy  (Baseline sample) | Accuracy (GAN sample) |
| --- | --- | --- | --- | --- | --- |
| nn1 | 20,10 | 64-1000 | 20,10 | 1.00 | 0.64 |
| nn2 | 20,10,5 | 64-1000 | 20,10 | 0.97 | 0.61 |
| nn3 | 20,40,10 | 64-1000 | 20,10 | 1.00 | 0.55 |
| nn4 | 30,15,5 | 64-1000 | 20,10 | 1.00 | 0.61 |
| nn5 | 60,30 | 64-1000 | 20,10 | 1.00 | 0.63 |
| Model14 (EL) |  | 64-1000 | 20,10 | **1.00 (F1=1.00)** | **0.71 (F1=0.70)** |

**Graphic display of cut marks**

In addition to the statistical analyses and their graphic display in the main text, we also include here the complete distribution of cut marks on 3D templates of each of the five meat-bearing bones analyzed with Ikhnos (Figures S7 and S8). Intensity of cut marks is most abundant in primary access experiments, because they reflect bulk defleshing, compared to just scrap removal in secondary access experiments. Cut marks are also more abundant in the primary access scenario compared to the archaeological samples because of two important factors. One is that the experiments are based on 100% preservation of all bones, either because they are complete (*Cervus elaphus* subsample) or because the smallest fragments resulting from demarrowing have also been preserved (*Ovis aries* subsample). In contrast, the archaeological long bone samples analyzed here do not preserved complete elements or all the fragments belonging to the same original bones. Postdepositional loss caused by carnivore deletion, potential vulture and small carnivore intervention (moving fragments away from the excavated areas), and the sedimentary cycle involving low-energy hydraulic processes may in combination have resulted in the loss of several bone fragments. Carnivore impact, although minor in terms of bone surface modifications, may have been more significant if considering density-mediated long bone portion preservation, which affects some of the portions that bear most cut marks when butchery is performed on complete fleshed carcasses. As an additional cause, it can be observed that in the primary access experiments that most cut marks concentrate on stylopodials, which not only are most affected by preservation processes, but also exhibit a higher concentration of additional marks imparted during systematic dismembering (as documented on epiphyseal portions). The archaeofaunal assemblages coincide in displaying very few (if any) cut marks on these portions caused by secondary dismembering (i.e., disarticulation of long bone elements), as opposed to what happens in modern foragers´camps. This may potentially be a reflection of the socio-reproductive behavior of early hominins compared to modern humans[(*54*)](https://paperpile.com/c/og77al/1B10n).


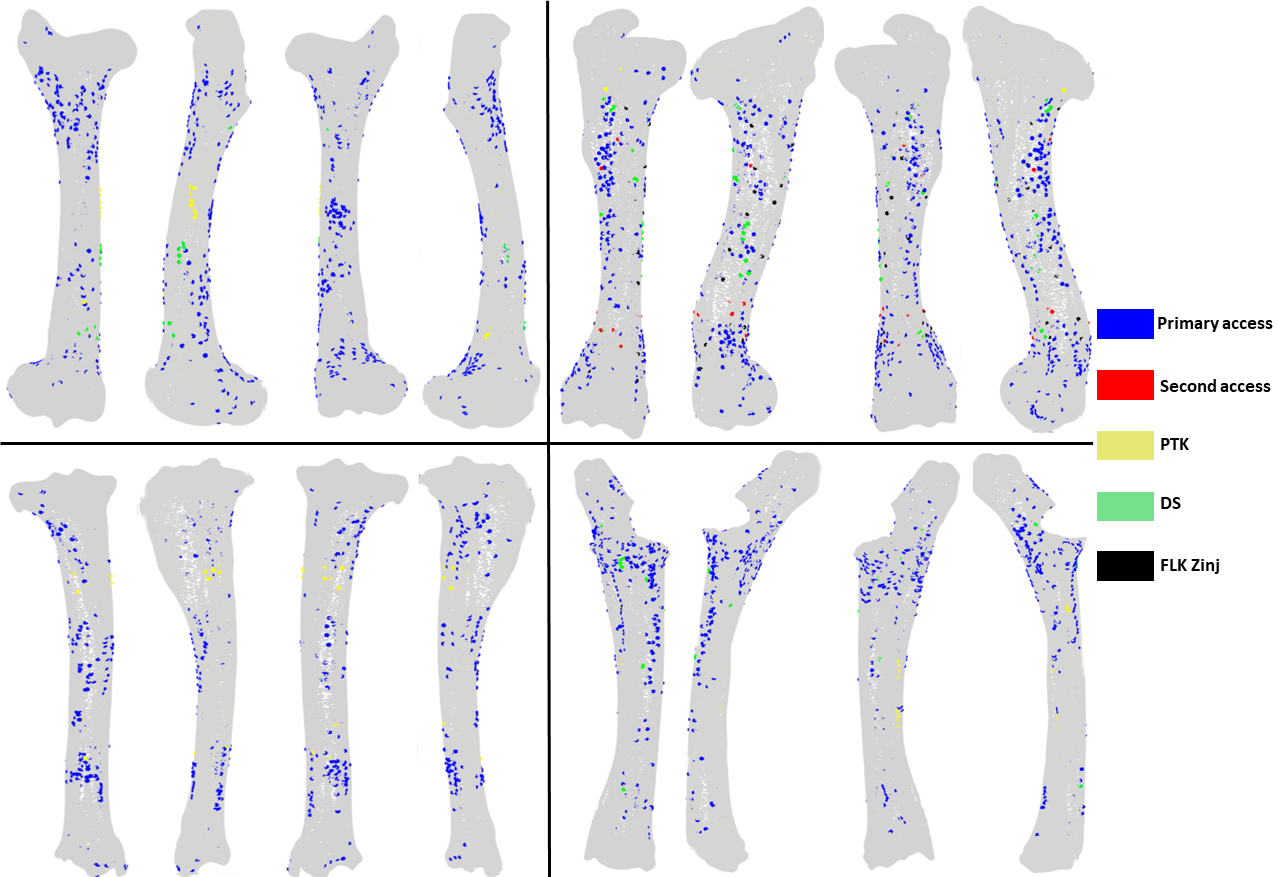


Figure S7. Display of cut marks on the 3D templates for each right meat-bearing long bone showing the exact occurrence of cut marks in both experimental scenarios and on the archaeological samples. Not all marks may be visible because of overlay of different experimental and archaeological assemblages. Figure created with Ikhnos.


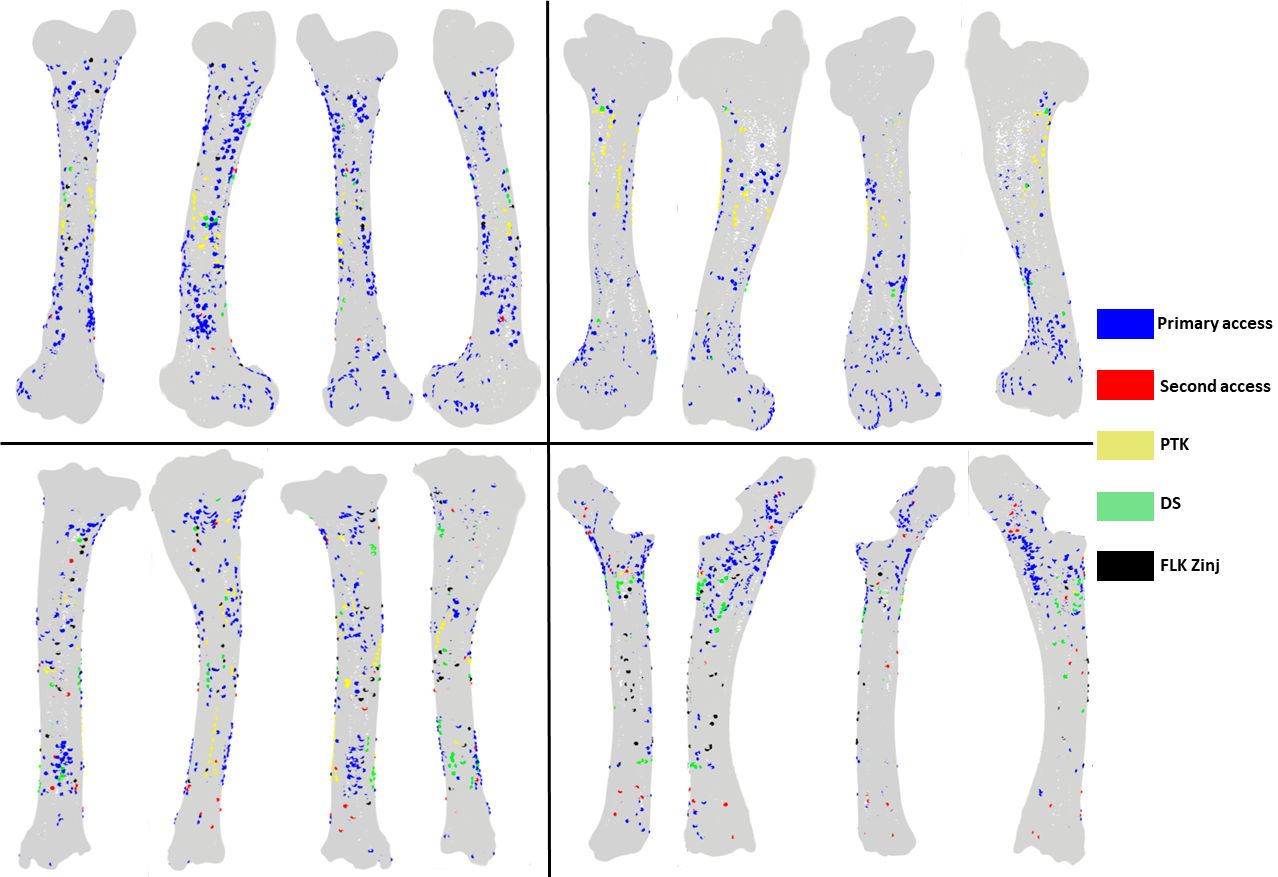


Figure S8. Display of cut marks on the 3D templates for each left meat-bearing long bone showing the exact occurrence of cut marks in both experimental scenarios and on the archaeological samples. Not all marks may be visible because of overlay of different experimental and archaeological assemblages. Figure created with Ikhnos.

**References**

1. [A. L. Deino, 40Ar/39Ar dating of Bed I, Olduvai Gorge, Tanzania, and the chronology of early Pleistocene climate change. *J. Hum. Evol.* **63**, 251–273 (2012).](http://paperpile.com/b/og77al/Dh71K)

2. [D. M. Martín-Perea, O. Fesharaki, J. J. Rey Samper, X. Arroyo, D. Uribelarrea, L. Cobo-Sánchez, E. Baquedano, A. Mabulla, M. Domínguez-Rodrigo, Mineral assemblages and low energy sedimentary processes in the FLK-Zinj, DS, PTK and AMK complex palaeolandscape (Olduvai Gorge, Tanzania). *Quat. Int.* **526**, 15–25 (2019).](http://paperpile.com/b/og77al/3dAcT)

3. [M. Domínguez-Rodrigo, A. J. Sánchez-Flores, Constraining time and ecology on the Zinj paleolandscape: Microwear and mesowear analyses of the archaeofaunal remains of FLK Zinj and DS (Bed I), compared to …. *Quaternary* (2019) (available at](http://paperpile.com/b/og77al/RdoFm) <https://www.sciencedirect.com/science/article/pii/S1040618219303209>[).](http://paperpile.com/b/og77al/RdoFm)

4. [H. T. Bunn, E. M. Kroll, S. H. Ambrose, A. K. Behrensmeyer, L. R. Binford, R. J. Blumenschine, R. G. Klein, H. M. McHenry, C. J. O’Brien, J. J. Wymer, Systematic Butchery by Plio/Pleistocene Hominids at Olduvai Gorge, Tanzania [and Comments and Reply]. *Curr. Anthropol.* **27**, 431–452 (1986).](http://paperpile.com/b/og77al/pIoQK)

5. [J. S. Oliver, Estimates of hominid and carnivore involvement in the FLK Zinjanthropus fossil assemblage: some socioecological implications. *J. Hum. Evol.* **27**, 267–294 (1994).](http://paperpile.com/b/og77al/YTMZY)

6. [R. J. Blumenschine, *Philos. Trans. R. Soc. Lond. B Biol. Sci.*, in press.](http://paperpile.com/b/og77al/H11Bs)

7. [M. Domínguez-Rodrigo, R. Barba, C. P. Egeland, *Deconstructing Olduvai: A Taphonomic Study of the Bed I Sites* (Springer Science & Business Media, 2007).](http://paperpile.com/b/og77al/EwD6D)

8. [M. C. Pante, R. J. Blumenschine, S. D. Capaldo, R. S. Scott, Validation of bone surface modification models for inferring fossil hominin and carnivore feeding interactions, with reapplication to FLK 22, Olduvai Gorge, Tanzania. *J. Hum. Evol.* **63**, 395–407 (2012).](http://paperpile.com/b/og77al/7Qj9N)

9. [J. A. Parkinson, *A GIS image analysis approach to documenting Oldowan hominin carcass acquisition: Evidence from Kanjera South, FLK Zinj, and neotaphonomic models of carnivore bone destruction* (City University of New York, 2013).](http://paperpile.com/b/og77al/zvQQ9)

10. [M. Domínguez-Rodrigo, H. T. Bunn, J. Yravedra, A critical re-evaluation of bone surface modification models for inferring fossil hominin and carnivore interactions through a multivariate approach: application to the FLK Zinj archaeofaunal assemblage (Olduvai Gorge, Tanzania). *Quat. Int.* **322**, 32–43 (2014).](http://paperpile.com/b/og77al/T5t3R)

11. [M. Domínguez-Rodrigo, L. Cobo-Sánchez, A spatial analysis of stone tools and fossil bones at FLK Zinj 22 and PTK I (Bed I, Olduvai Gorge, Tanzania) and its bearing on the social organization of early humans. *Palaeogeogr. Palaeoclimatol. Palaeoecol.* **488**, 21–34 (2017).](http://paperpile.com/b/og77al/t3YeF)

12. [M. Domínguez-Rodrigo, A. J. Sánchez-Flores, E. Baquedano, M. C. Arriaza, J. Aramendi, L. Cobo-Sánchez, E. Organista, R. Barba, Constraining time and ecology on the Zinj paleolandscape: Microwear and mesowear analyses of the archaeofaunal remains of FLK Zinj and DS (Bed I), compared to FLK North (Bed I) and BK (Bed II) at Olduvai Gorge (Tanzania). *Quat. Int.* **526**, 4–14 (2019).](http://paperpile.com/b/og77al/U8pfO)

13. [M. Domínguez-Rodrigo, E. Baquedano, R. Barba, D. Uribelarrea, A. Gidna, The river that never was: Fluvial taphonomy at Olduvai Bed I and II sites and its bearing on early human behavior. *Quat. Int.* **526**, 26–38 (2019).](http://paperpile.com/b/og77al/jxQbs)

14. [M. Domínguez-Rodrigo, L. Cobo-Sánchez, J. Aramendi, A. Gidna, The meta-group social network of early humans: A temporal–spatial assessment of group size at FLK Zinj (Olduvai Gorge, Tanzania). *J. Hum. Evol.* **127**, 54–66 (2019).](http://paperpile.com/b/og77al/EyXgC)

15. [D. Uribelarrea, M. Domínguez-Rodrigo, A. Pérez-González, J. Vegas Salamanca, E. Baquedano, A. Mabulla, C. Musiba, D. Barboni, L. Cobo-Sánchez, Geo-archaeological and geometrically corrected reconstruction of the 1.84 Ma FLK Zinj paleolandscape at Olduvai Gorge, Tanzania. *Quat. Int.* **322-323**, 7–31 (2014).](http://paperpile.com/b/og77al/cZXXH)

16. [M. D. Leakey, *Olduvai Gorge. Vol. 3, Excavations in Beds I and II, 1960-63* (Cambridge University Press, 1971).](http://paperpile.com/b/og77al/V66yk)

17. [H. Arráiz, D. Barboni, G. M. Ashley, A. Mabulla, E. Baquedano, M. Domínguez-Rodrigo, The FLK Zinj paleolandscape: Reconstruction of a 1.84Ma wooded habitat in the FLK Zinj-AMK-PTK-DS archaeological complex, Middle Bed I (Olduvai Gorge, Tanzania). *Palaeogeogr. Palaeoclimatol. Palaeoecol.* **488**, 9–20 (2017).](http://paperpile.com/b/og77al/nYyci)

18. [M. Domínguez-Rodrigo, R. Barba, A Study of Cut Marks on Small-Sized Carcasses and its Application to the Study of Cut-Marked Bones from Small Mammals at the FLK Zinj Site. *Blumenschine*. **1988**, 1995 (1991).](http://paperpile.com/b/og77al/Qlk7s)

19. [A. B. Galán, M. Domínguez-Rodrigo, An Experimental Study of the Anatomical Distribution of Cut Marks Created by Filleting and Disarticulation on Long Bone Ends. *Archaeometry*. **55**, 1132–1149 (2013).](http://paperpile.com/b/og77al/Cd6lE)

20. [A. O. Gidna, B. Kisui, A. Mabulla, C. Musiba, M. Domínguez-Rodrigo, An ecological neo-taphonomic study of carcass consumption by lions in Tarangire National Park (Tanzania) and its relevance for human evolutionary biology. *Quat. Int.* **322–323**, 167–180 (2014).](http://paperpile.com/b/og77al/nNAJ0)

21. [M. Pizarro-Monzo, M. E. Prendergast, A. O. Gidna, E. Baquedano, R. Mora, D. Gonzalez-Aguilera, M. A. Mate-Gonzalez, M. Domínguez-Rodrigo, Do human butchery patterns exist? A study of the interaction of randomness and channelling in the distribution of cut marks on long bones. *J. R. Soc. Interface*. **18**, 20200958 (2021).](http://paperpile.com/b/og77al/vEbZ9)

22. [R. L. Lyman, Archaeofaunas and Butchery Studies: A Taphonomic Perspective. *Advances in Archaeological Method and Theory*. **10**, 249–337 (1987).](http://paperpile.com/b/og77al/8JHHp)

23. [R. L. Lyman, A study of variation in the prehistoric butchery of large artiodactyls. *Ancient peoples and landscapes*, 233–253 (1995).](http://paperpile.com/b/og77al/z5tZe)

24. [I. Goodfellow, J. Pouget-Abadie, M. Mirza, B. Xu, D. Warde-Farley, S. Ozair, A. Courville, Y. Bengio, in *Advances in Neural Information Processing Systems*, Z. Ghahramani, M. Welling, C. Cortes, N. Lawrence, K. Q. Weinberger, Eds. (Curran Associates, Inc., 2014), vol. 27, pp. 2672–2680.](http://paperpile.com/b/og77al/pwQ6V)

25. [K. Lata, M. Dave, K. N. Nishanth, Data Augmentation Using Generative Adversarial Network. *SSRN Electronic Journal*, , doi:](http://paperpile.com/b/og77al/YzF1R)[10.2139/ssrn.3349576](http://dx.doi.org/10.2139/ssrn.3349576)[.](http://paperpile.com/b/og77al/YzF1R)

26. [L. Yi, M.-W. Mak, Improving Speech Emotion Recognition With Adversarial Data Augmentation Network. *IEEE Trans Neural Netw Learn Syst*. **PP** (2020), doi:](http://paperpile.com/b/og77al/OrR2z)[10.1109/TNNLS.2020.3027600](http://dx.doi.org/10.1109/TNNLS.2020.3027600)[.](http://paperpile.com/b/og77al/OrR2z)

27. [D. Yorioka, H. Kang, K. Iwamura, Data Augmentation For Deep Learning Using Generative Adversarial Networks. *2020 IEEE 9th Global Conference on Consumer Electronics (GCCE)* (2020), , doi:](http://paperpile.com/b/og77al/g0bCa)[10.1109/gcce50665.2020.9291963](http://dx.doi.org/10.1109/gcce50665.2020.9291963)[.](http://paperpile.com/b/og77al/g0bCa)

28. [Y. Tang, S. Oh, J. Xiao, R. M. Summers, Y. Tang, CT-realistic data augmentation using generative adversarial network for robust lymph node segmentation. *Medical Imaging 2019: Computer-Aided Diagnosis* (2019), , doi:](http://paperpile.com/b/og77al/6OLLC)[10.1117/12.2512004](http://dx.doi.org/10.1117/12.2512004)[.](http://paperpile.com/b/og77al/6OLLC)

29. [Y. Sun, P. Yuan, Y. Sun, MM-GAN: 3D MRI Data Augmentation for Medical Image Segmentation via Generative Adversarial Networks. *2020 IEEE International Conference on Knowledge Graph (ICKG)* (2020), , doi:](http://paperpile.com/b/og77al/ysQ7r)[10.1109/icbk50248.2020.00041](http://dx.doi.org/10.1109/icbk50248.2020.00041)[.](http://paperpile.com/b/og77al/ysQ7r)

30. [Y.-J. Lin, I.-F. Chung, Medical Data Augmentation Using Generative Adversarial Networks : X-ray Image Generation for Transfer Learning of Hip Fracture Detection. *2019 International Conference on Technologies and Applications of Artiﬁcial Intelligence (TAAI)* (2019), , doi:](http://paperpile.com/b/og77al/i8Pbl)[10.1109/taai48200.2019.8959908](http://dx.doi.org/10.1109/taai48200.2019.8959908)[.](http://paperpile.com/b/og77al/i8Pbl)

31. [M. Podduturi, Data augmentation for supervised learning with generative adversarial networks, , doi:](http://paperpile.com/b/og77al/6asE9)[10.31274/etd-180810-6069](http://dx.doi.org/10.31274/etd-180810-6069)[.](http://paperpile.com/b/og77al/6asE9)

32. [D. A. van Dyk, X.-L. Meng, The Art of Data Augmentation. *J. Comput. Graph. Stat.* **10**, 1–50 (2001).](http://paperpile.com/b/og77al/4Stvk)

33. [V. Sandfort, K. Yan, P. J. Pickhardt, R. M. Summers, Data augmentation using generative adversarial networks (CycleGAN) to improve generalizability in CT segmentation tasks. *Sci. Rep.* **9**, 16884 (2019).](http://paperpile.com/b/og77al/Ltara)

34. [G. St-Yves, T. Naselaris, Generative Adversarial Networks Conditioned on Brain Activity Reconstruct Seen Images, , doi:](http://paperpile.com/b/og77al/VEtXM)[10.1101/304774](http://dx.doi.org/10.1101/304774)[.](http://paperpile.com/b/og77al/VEtXM)

35. [O. Takeuchi, H. Shishido, Y. Kameda, H. Kim, I. Kitahara, Image-quality Improvement of Omnidirectional Free-viewpoint Images by Generative Adversarial Networks. *Proceedings of the 15th International Joint Conference on Computer Vision, Imaging and Computer Graphics Theory and Applications* (2020), , doi:](http://paperpile.com/b/og77al/hG41F)[10.5220/0008959802990306](http://dx.doi.org/10.5220/0008959802990306)[.](http://paperpile.com/b/og77al/hG41F)

36. [S. Kurupathi, P. Murthy, D. Stricker, Generation of Human Images with Clothing using Advanced Conditional Generative Adversarial Networks. *Proceedings of the 1st International Conference on Deep Learning Theory and Applications* (2020), , doi:](http://paperpile.com/b/og77al/H6EqE)[10.5220/0009832200300041](http://dx.doi.org/10.5220/0009832200300041)[.](http://paperpile.com/b/og77al/H6EqE)

37. [S. Guan, M. Loew, Evaluation of Generative Adversarial Network Performance Based on Direct Analysis of Generated Images. *2019 IEEE Applied Imagery Pattern Recognition Workshop (AIPR)* (2019), , doi:](http://paperpile.com/b/og77al/8Z9k5)[10.1109/aipr47015.2019.9174595](http://dx.doi.org/10.1109/aipr47015.2019.9174595)[.](http://paperpile.com/b/og77al/8Z9k5)

38. [F. A. Faria, G. Carneiro, Why are Generative Adversarial Networks so Fascinating and Annoying? *2020 33rd SIBGRAPI Conference on Graphics, Patterns and Images (SIBGRAPI)* (2020), , doi:](http://paperpile.com/b/og77al/VuPGP)[10.1109/sibgrapi51738.2020.00009](http://dx.doi.org/10.1109/sibgrapi51738.2020.00009)[.](http://paperpile.com/b/og77al/VuPGP)

39. [L. Xu, M. Skoularidou, A. Cuesta-Infante, K. Veeramachaneni, Modeling Tabular data using Conditional GAN. *arXiv [cs.LG]* (2019), (available at](http://paperpile.com/b/og77al/jwgT0) <http://arxiv.org/abs/1907.00503>[).](http://paperpile.com/b/og77al/jwgT0)

40. [M. Arjovsky, S. Chintala, L. Bottou, in *International conference on machine learning* (PMLR, 2017), pp. 214–223.](http://paperpile.com/b/og77al/lAPef)

41. [P. Schäfer, Scalable time series classification. *Data Min. Knowl. Discov.* **30**, 1273–1298 (2016).](http://paperpile.com/b/og77al/rmBMv)

42. [P. Senin, S. Malinchik, in *2013 IEEE 13th International Conference on Data Mining* (2013), pp. 1175–1180.](http://paperpile.com/b/og77al/7flAN)

43. [J. Lin, R. Khade, Y. Li, Rotation-invariant similarity in time series using bag-of-patterns representation. *J. Intell. Inf. Syst.* **39**, 287–315 (2012).](http://paperpile.com/b/og77al/IMxl4)

44. [P. Schäfer, U. Leser, in *Proceedings of the 2017 ACM on Conference on Information and Knowledge Management* (Association for Computing Machinery, New York, NY, USA, 2017), *CIKM ’17*, pp. 637–646.](http://paperpile.com/b/og77al/Ynddz)

45. [Z. Wang, T. Oates, in *Workshops at the twenty-ninth AAAI conference on artificial intelligence* (2015;](http://paperpile.com/b/og77al/Ik8ot) <https://www.researchgate.net/profile/Zhiguang_Wang3/publication/275970614_Encoding_Time_Series_as_Images_for_Visual_Inspection_and_Classification_Using_Tiled_Convolutional_Neural_Networks/links/554ceb960cf21ed2135f5951.pdf>[), vol. 1.](http://paperpile.com/b/og77al/Ik8ot)

46. [M. Domı́nguez-Rodrigo, Meat-eating by early hominids at the FLK 22Zinjanthropussite, Olduvai Gorge (Tanzania): an experimental approach using cut-mark data. *J. Hum. Evol.* **33**, 669–690 (1997).](http://paperpile.com/b/og77al/DTP4N)

47. [L. A. Courtenay, D. González-Aguilera, Geometric morphometric data augmentation using generative computational learning algorithms. *Appl. Sci.* **10**, 9133 (2020).](http://paperpile.com/b/og77al/lt10q)

48. [A. Kumar, M. Jain, Using Ensemble Learning Libraries. *Ensemble Learning for AI Developers* (2020), pp. 61–96.](http://paperpile.com/b/og77al/Yh2F2)

49. [G. Kyriakides, K. G. Margaritis, *Hands-On Ensemble Learning with Python: Build highly optimized ensemble machine learning models using scikit-learn and Keras* (Packt Publishing Ltd, 2019).](http://paperpile.com/b/og77al/SzItQ)

50. [P. N. Tattar, *Hands-On Ensemble Learning with R: A beginner’s guide to combining the power of machine learning algorithms using ensemble techniques* (Packt Publishing Ltd, 2018).](http://paperpile.com/b/og77al/5xvT6)

51. [D. H. Wolpert, Stacked generalization. *Neural Netw.* **5**, 241–259 (1992).](http://paperpile.com/b/og77al/hHcON)

52. [D. H. Wolpert, The Existence of A Priori Distinctions Between Learning Algorithms. *Neural Comput.* **8**, 1391–1420 (1996).](http://paperpile.com/b/og77al/0Zk6O)

53. [J. Brownlee, *Machine Learning Mastery With R: Get Started, Build Accurate Models and Work Through Projects Step-by-Step* (Machine Learning Mastery, 2016).](http://paperpile.com/b/og77al/iN32R)

54. [M. Domínguez-Rodrigo, L. Cobo-Sánchez, The spatial patterning of the social organization of modern foraging Homo sapiens: A methodological approach for understanding social organization in prehistoric foragers. *Palaeogeogr. Palaeoclimatol. Palaeoecol.* **488**, 113–125 (2017).](http://paperpile.com/b/og77al/1B10n)
